# Safety and effectiveness of mass drug administration to accelerate elimination of artemisinin-resistant falciparum malaria: A pilot trial in four villages of Eastern Myanmar

**DOI:** 10.12688/wellcomeopenres.12240.1

**Published:** 2017-09-06

**Authors:** Jordi Landier, Ladda Kajeechiwa, May Myo Thwin, Daniel M. Parker, Victor Chaumeau, Jacher Wiladphaingern, Mallika Imwong, Olivo Miotto, Krittaya Patumrat, Jureeporn Duanguppama, Dominique Cerqueira, Benoit Malleret, Laurent Rénia, Suphak Nosten, Lorenz von Seidlein, Clare Ling, Stéphane Proux, Vincent Corbel, Julie A. Simpson, Arjen M. Dondorp, Nicholas J. White, François H. Nosten

**Affiliations:** 1 Shoklo Malaria Research Unit, Mahidol-Oxford Tropical Medicine Research Unit, Faculty of Tropical Medicine, Mahidol University, Mae Sot, Thailand; 2 Maladies Infectieuses et Vecteurs Ecologie, Génétique, Evolution et Contrôle (IRD 224-CNRS 4280 UM1-UM2), Institut de Recherche pour le Développement, Montpellier, France; 3 Centre Hospitalier Régional Universitaire de Montpellier, Montpellier, France; 4 Mahidol Oxford Research Unit, Faculty of Tropical Medicine, Mahidol University, Bangkok, Thailand; 5 Department of Molecular Tropical Medicine and Genetics, Faculty of Tropical Medicine, Mahidol University, Bangkok, Thailand; 6 Centre for Genomics and Global Health, Wellcome Trust Centre for Human Genetics, University of Oxford, Oxford, OX3 7BN, UK; 7 Wellcome Trust Sanger Institute, Cambridge, CB10 1SA, UK; 8 Department of Microbiology & Immunology, Yong Loo Lin School of Medicine, National University of Singapore, Singapore, 119077, Singapore; 9 Singapore Immunology Network (SIgN), Agency for Science & Technology, Singapore, 138632, Singapore; 10 Centre for Epidemiology and Biostatistics, Melbourne School of Population and Global Health, The University of Melbourne, Melbourne, VIC, 3053, Australia; 11 Centre for Tropical Medicine and Global Health, Nuffield Department of Medicine, University of Oxford, Oxford, OX3 7BN, UK

**Keywords:** Plasmodium falciparum, Plasmodium vivax, Mass Drug Administration, Asymptomatic carriage, submicroscopic infection, artemisinin-resistance, transmission

## Abstract

**Background: **Artemisinin and partner drug-resistant falciparum malaria is expanding over the Greater Mekong Sub-region (GMS). Eliminating falciparum malaria in the GMS while drugs still retain enough efficacy could prevent global spread of antimalarial resistance. Eliminating malaria rapidly requires targeting the reservoir of asymptomatic parasite carriers.

This pilot trial aimed to evaluate the acceptability, safety, feasibility and effectiveness of mass-drug administration (MDA) in reducing malaria in four villages in Eastern Myanmar.

**Methods: **Villages with ≥30% malaria prevalence were selected. Long-lasting insecticidal bednets (LLINs) and access to malaria early diagnosis and treatment (EDT) were provided. Two villages received MDA immediately and two were followed for nine months pre-MDA. MDA consisted of a 3-day supervised course of  dihydroartemisinin-piperaquine and single low-dose primaquine administered monthly for three months. Adverse events (AE) were monitored by interviews and consultations. Malaria prevalence was assessed by ultrasensitive PCR quarterly for 24 months. Symptomatic malaria incidence,entomological indices, and antimalarial resistance markers were monitored.

**Results: **MDA was well tolerated. There were no serious AE and mild to moderate AE were reported in 5.6%(212/3931) interviews. In the smaller villages, participation to three MDA courses was 61% and 57%, compared to 28% and 29% in the larger villages. Baseline prevalence was higher in intervention than in control villages (18.7% (95%CI=16.1-21.6) versus 6.8%(5.2-8.7), p<0.0001) whereas three months after starting MDA, prevalence was lower in intervention villages (0.4%(0.04-1.3) versus 2.7%(1.7-4.1), p=0.0014). After nine months the difference was no longer significant (2.0%(1.0-3.5) versus 0.9%(0.04-1.8), p=0.10). M0-M9 symptomatic falciparum incidence was similar between intervention and control. Before/after MDA comparisons showed that asymptomatic *P. falciparum *carriage and anopheline vector positivity decreased significantly whereas prevalence of the artemisinin-resistance molecular marker remained stable.

**Conclusions: **This MDA was safe and feasible, and, could accelerate elimination of *P. falciparum *in addition to EDT and LLINs **when community participation was sufficient.

## Introduction

Recent progress in the control of malaria is now under threat because the two pillars of malaria control – antimalarial drugs and insecticides – are falling to resistance ^1^. Historically, the Greater Mekong sub–region (GMS) has been the source of antimalarial drug resistance, and artemisinin resistant *Plasmodium falciparum* has also emerged and recently spread in this region ^2^. Artemisinin resistance has led to the failure of artemisinin based combination treatments (ACT) ^3, 4^, the cornerstone of uncomplicated *P. falciparum* malaria therapy worldwide. Without alternative medicines in the near future, the best option to prevent the spread of resistant parasites to Africa is the rapid elimination of *P. falciparum* parasites from the GMS. Early detection and treatment of clinical cases at the community level is effective in preventing mortality and reduces *P. falciparum* transmission and the associated morbidity ^5– 7^. However this approach alone is unlikely to halt the rapid spread of artemisinin resistance because it fails to directly address foci of asymptomatic carriage of *P. falciparum*. This persistent reservoir represents the main obstacle to the rapid elimination of falciparum malaria in the GMS, where transmission is low, seasonal and unstable ^8, 9^.

Systematic antimalarial drug treatment of the entire population of these foci may be the only method to achieve a rapid elimination of the parasite reservoir in the absence of a point-of-care test sensitive enough to allow mass screening and treatment of submicroscopic carriers ^10– 12^. Mass drug administration (MDA) in malaria remains controversial because of its history of success and failure ^13^. MDA in the ACT era often relies on piperaquine, which provides approximately a one-month protection from reinfection. Adding a single low-dose of primaquine rapidly sterilises *P. falciparum* infections ^14^. Promising short term results following MDA were recently obtained in Zambia, albeit in a context of generally decreasing malaria prevalence ^15^. We present a 24-month pilot study of the safety and effectiveness of MDA in reducing *P. falciparum* incidence and prevalence in four villages with high prevalence of sub-microscopic infections located on the Thailand–Myanmar border, an area where artemisinin resistance is firmly established ^16, 17^.

## Methods

### Study design

This study was the pilot phase of a multicentre cluster-randomized control trial conducted in several sites in the GMS and therefore limited to a sample size of four villages. It is registered at ClinicalTrials.gov: NCT01872702.

The study was conducted in rural Eastern Kayin (Karen) state of Myanmar, a region endemic for *P. falciparum* and *P. vivax* malaria ^17^. In early 2013, 12 villages were screened using a high volume ultra-sensitive qPCR assay method (uPCR) with the original intention of identifying villages with >30% malaria prevalence, of which >30% was *P. falciparum* ( Supplementary File 1: Table S1). However, the *P. falciparum* prevalence was lower than expected, so after the survey the threshold proportion of *P. falciparum* for inclusion was lowered to 10%. Four villages fulfilling this revised entry criterion were selected after community engagement and agreement by the village leaders. A malaria post (MP) was set up at month 0 (M0) in all four villages and long lasting insecticidal nets (LLIN) were provided. Two villages (referred to as A1-KNH and A2-TOT) were randomized using a flipped coin to receive anti-malarial MDA for three consecutive months (M0 to M2) immediately. The other two villages (referred to as B1-TPN and B2-HKT) were monitored for a 9-month control period with MP and LLIN only. The control period was planned originally for 12 months, but MDA had to be expedited because of accessibility concerns during the rainy season, and was given from M9 to M11. All four villages were followed for 24 months in total.

### Ethics statement

This study protocol was reviewed and approved by OxTREC (reference no. 1017–13 and 1015–13), by the Tak Community Advisory Board ^18^, and by village committees. Participation in surveys and MDA activities was voluntary. In addition to group information during community engagement activities, participants received individual information in their language (Karen or Myanmar) and provided written informed consent before inclusion in a survey or MDA. Participants under 18 years provided an assent in addition to the consent of their parent or guardian.

### Study participants

Participants were defined as individuals living in the village who provided information during home visits at any time point. Each participant was followed using a unique identification code to collect information on participation, adverse events, malaria carriage during surveys, clinical episodes of malaria, and mobility.

### Interventions

Before and during the study, community engagement (CE) activities were conducted (detailed in 19). Briefly, the aim of community engagement was to present the project to the community and involve its members to create understanding and buy-in of the population, and result in a high coverage of the interventions ^20^. The different activities conducted were: workshops with authorities and local gatekeepers, meetings and activities with different population groups, health-related community incentives (ancillary care and health education; building of water catchment and distribution systems in each of the study villages) ^19^. Through this mutual understanding, the study activities could be conducted while respecting the life of the community and ensuring maximal benefit for the participants.

A population census was conducted, individuals were linked to houses (with both individual and house identification codes), and geographic references (latitude and longitude) were recorded for each house. Population movement in and out of villages was monitored by census during the quarterly surveys and by home visits. Newcomers intending to stay in the village for more than two weeks were enrolled after consenting to participate in the follow-up surveys. All newcomers arriving after MDA were offered a single curative treatment course of DP-primaquine, irrespective of disease or infection status.

A MP was set up at M0 in all four villages to provide early detection and treatment of clinical cases ^7^. Fever cases were tested with SD Bioline Pf/Pv rapid diagnostic test (RDT). Uncomplicated *P. falciparum* cases were given weight-adjusted doses of dihydroartemisinin-piperaquine (DP, Guilin Pharmaceutical Co, Guilin, PRC) for three days and one 0.25mg base/kg dose of primaquine (Governement Pharmaceutical Organization, Thailand). Uncomplicated *P. vivax* cases ** were given weight-adjusted doses of chloroquine (Governement Pharmaceutical Organization, Thailand) for three days. Severe illnesses were referred to the nearest clinic. LLINs were distributed to all households at M0.

Blood sampling surveys of the entire village population were conducted at months 0, 3, 6, 9, 12, 15, 18, and 24. Samples of 3 and 0.5 mL of blood were taken by venous puncture from survey participants over and under 5 years respectively. Malaria infection was detected in the laboratory by RDT, microscopy and by a uPCR method with an approximate limit of detection of 20 parasites/mL ^8, 21^.

Each MDA comprised a standard 3–day treatment course of DP (dihydroartemisinin (7mg/kg) and piperaquine (55 mg/kg) and one 0.25mg base/kg dose of primaquine given orally under supervision. Treatments were given three times at one-month intervals (M0 to M2 or M9 to M11).

### Inclusion and exclusion criteria

Infants <6 months, individuals with a known allergy to any of the antimalarial drugs, and pregnant women in their first trimester were excluded from MDA participation. Pregnant women in their second or third trimester and breastfeeding mothers were eligible for DP, but excluded from primaquine treatment. Women of childbearing age were asked if they were or could be pregnant and a pregnancy test was offered if unsure. Infants <6 months were excluded from prevalence surveys.

### Adverse event monitoring

Adverse events (AE) were monitored using a structured questionnaire on the second, third, and seventh day after the start of the MDA treatment course. During the three months of MDA, a mobile clinic staffed with a medical assistant was available in the village to provide free consultations to all villagers and to assess MDA participants presenting with AE. Serious adverse events were reported centrally.

### Variable collection and definition

***Participant follow-up***. The individual follow-up time was defined as the number of days spent within the catchment area of the MP, i.e. the village and the surrounding farms.

***Participation in MDA***. Participation in MDA was measured as the total number of individuals completing one, two or three 3-day DP treatment courses divided by the total number of inhabitants present in the village at least once during the 3-month period, after excluding individuals who visited the village for two weeks or less. Individual participation in MDA was categorized in three groups: no MDA, one or two rounds (i.e. infection cured, but incomplete protection from reinfection by DP), and three rounds MDA (i.e. infection cured and 3-month protection).

***Malaria infection prevalence***. Prevalence of *P. falciparum* or *P. vivax* infection was defined as the proportion of individuals with a positive uPCR for each parasite divided by total number of individuals sampled. When the parasite species could not be determined (because of low DNA content) samples were attributed to species based on the falciparum/vivax ratio measured in clinical cases in the village during the previous period ^8^.

***Incidence of symptomatic malaria***. Symptomatic malaria cases were defined as individuals with fever (temperature ≥37.5°C) or history of fever in the past two days and confirmed falciparum or vivax infection by RDT or microscopy. During the period between surveys, cases were detected passively at the MP and confirmed by RDT. During survey periods, individuals with fever or history of fever and a positive RDT or microscopy slide (with uPCR confirmation when available) were considered clinical cases. Incidence of clinical malaria episodes in the village was expressed in cases per 1000 persons per month (number of infections during the month/sum of individual follow-up time during the month). Patients residing in nearby villages and coming to the MP for diagnosis were excluded from the analysis. Mixed infections contributed to both *P. falciparum* and *P. vivax* incidence.

### Detection of molecular markers of antimalarial resistance

***Assessment of mutations in PfKelch13***. Polymorphisms in the *PfKelch* gene were assessed by nested PCR amplification covering the full length of the gene (total 2181 bp) ^2^, and followed by sequencing of the gene by ABI Sequencer (Macrogen Inc, South Korea). Cross contamination was monitored by adding negative control samples in every run. Sequencing results were aligned against *PfKelch13* of reference strain 3D7 (putative 9PF13_0238 NCBI Reference Sequence (3D7): XM_001350122.1), using Bioedit software (Abbott, CA, USA). Polymorphic patterns were assessed by two individuals blinded to the origin of the sample.

***PfPlasmepsin2 gene amplification***. *Pfplasmepsin2* copy number was quantified using Relative-quantitative Real-time PCR based on Taqman probe on a Corbett Rotor-Gene ^TM^ Q (Corbett Research, Australia). Primers and probes have been described previously ^22^. Amplification was performed in triplicate on a total volume of 25 μL as multiplex PCR using Quantitec Multiplex PCR no ROX (QIAgen, Germany). Every amplification run contained 9 replicates of calibrators and triplicates without template as negative controls. *β-tubulin* served as an internal standard for the amount of sample DNA added to the reactions. Copy numbers were calculated using the formula: copy number= **2 ^-ΔΔCt^** with **ΔΔ C _t_** denoting the difference between Δ C _t_ of the unknown sample and Δ C _t_ of the reference sample. ****

### Entomology

Entomological surveys to identify malaria vectors species and abundance were conducted monthly in each village for five nights per month, as described elsewhere ^23, 24^. Briefly, mosquitos were collected using indoor and outdoor human landing catch (HLC) in five sites per village, yielding a total of 50 human.nights per survey. Infection of primary malaria vectors ( *Anopheles minimus* *s.l.*, *An. maculatus* *s.l.*, and *An. dirus* *s.l.*) by plasmodium parasites was determined using a quantitative real-time PCR (qPCR) assay, confirmed with a second assay amplifying a different DNA target ^25^. The limit of detection of the whole procedure was 6 *P. falciparum* sporozoites per mosquito ^25^. Main entomological indices - human biting rate (HBR), sporozoite index (SI), and entomological inoculation rate (EIR) - were calculated.

### Statistical analysis

Statistical analyses were performed using Stata 14.1 (StataCorp, USA). Binomial 95% confidence intervals (CIs) were calculated for prevalence and SI, and Poisson 95% CIs for incidence of clinical episodes, HBR, and EIR. Intervention and control group prevalence were compared using chi-square or Fisher’s exact test, as appropriate.

### Pre-specified endpoints

Because the MDA in control villages was conducted earlier than originally planned (M9 instead of M12), the revised primary endpoints were defined as the prevalence of asymptomatic *P. falciparum* infection 3 and 9 months after MDA. The secondary endpoints were defined as 1) safety and 2) acceptability of MDA, as evaluated by questionnaires. Additional outcome measures reported here are the incidence of clinical malaria over the first 9 months, number of people participating in MDA, and proportion of artemisinin-resistant *P. falciparum* infections.

### Before/after analysis

A post-hoc before/after analysis of the entire duration of follow-up was conducted to understand the factors influencing the impact of antimalarial MDA and the overall intervention. The effect of the intervention on *P. falciparum* carriage (recorded in prevalence surveys) was estimated using Generalised Estimating Equations (GEE) with a logit link to estimate Odds Ratios (ORs), an exchangeable correlation structure and robust standard errors to account for repeated measurements at individual level. The change in odds of P. falciparum carriage compared to baseline survey was estimated in the control period and in the after MDA period. To assess the duration of MDA effect, time-dependent changes in the odds of *P. falciparum* carriage were estimated separately for the control and after MDA periods. Demographic characteristics, village, age group (≤ & >10 years), sex and season, were included as covariates. The analysis was repeated to assess the effect of individual MDA participation. A direct comparison of control and after MDA periods was also conducted in which baseline *P. falciparum* infection status was added as a covariate in addition to the demographic characteristics.

## Results

The study was conducted between May 2013 and June 2015 ( Table 1). Of 3238 individuals recorded in the villages during the 24-month study period, 2941 participated in surveys or MDA at least once ( Table 1). The population structure and mobility were typical of rural villages in this area ( Supplementary File 1: Figure S2). There was substantial turnover of the population in all villages: at any time point, between 19 and 27% of the population corresponded to individuals with <12 months follow-up ( Table 1, Supplementary File 1: Figure S2 and Figure S3). Around 70% of people stayed in the village continuously during the three consecutive months of MDA.

**Table 1.** Demographics, follow-up of participants, population coverage with mass drug administration (MDA) and duration of intervention, and access to malaria post of the four study villages in Eastern Kayin State, Myanmar. A map of the villages is presented in Supplementary File 1: Figure S1.

	A1–KNH	A2–TOT	B1–TPN	B2–HKT
***Village randomization information***				
Pilot survey *P. falciparum* prevalence: % (n/N)	16 (8/51)	7 (3/41)	4 (2/54)	15 (7/48)
Pilot survey malaria prevalence: % (n/N)	42 (22/51)	42 (17/41)	32 (17/54)	42 (20/48)
Randomization group	Intervention	Intervention	Control M0M9	Control M0M9
Study start date (M0)	12/06/2013	27/05/2013	20/05/2013	02/06/2013
Study end date (M24)	07/05/2015	25/04/2015	27/03/2015	04/06/2015
***Population and follow-up***				
Total population recorded over 24 months	494	940	488	1316
Participants over 24 months: n (%)	484 (98)	799 (85)	473 (97)	1185 (90)
Median age [IQR] (years)	23 [9–41]	19 [7–38]	20 [6–38]	19 [8–38]
Newcomers before MDA (n)	na	na	114	293
Newcomers during MDA (n)	83	75	7	112
Newcomers after MDA (n)	103	271	46	191
Newcomers taking 1 systematic treatment upon arrival, after MDA: n/N (%)	63/103 (61)	34/271 (13)	25/46 (54)	30/191 (16)
Prevalence of *P. falciparum* infection among newcomers after MDA: n/N (%)	2/78 (2.6)	3/150 (2.0)	0/32 (0)	1/170 (0.6)
***Average "real time" population composition: n (%)***				
Number of inhabitants ‡	344	610	317	837
Female ‡	163 (47)	301 (49)	147 (46)	395 (47)
Male ‡	181 (53)	309 (51)	170 (54)	442 (53)
Participants with >12 months follow-up ‡	277 (81)	463 (76)	257 (81)	613 (73)
Participants with 3–12 months follow-up ‡	58 (17)	124 (20)	51 (16)	192 (23)
Participants with <3 months ‡	9 (3)	23 (4)	9 (3)	32 (4)
***MDA implementation***				
MDA start date	12/06/2013	27/05/2013	28/01/2014	01/04/2014
Study month of MDA start	M0	M0	M9	M9
Duration of round 1; 2; 3 of MDA (days)	9; 10; 11	7; 14; 10	6; 9; 4	9; 9; 12
Duration to administer 3 doses to >90% of participants in 1 round: mean (days)	4	6	4	6
***Population during MDA period and MDA coverage: n (%)***				
Present at least once during the 3 MDA months	386 (100)	662 (100)	367 (100)	977 (100)
Present continuously during 3 MDA months	280 (73)	488 (74)	283 (77)	618 (63)
Taking 0 round of MDA	26 (7)	175 (26)	28 (8)	297 (30)
Taking 1 round of MDA	60 (16)	180 (27)	64 (17)	220 (23)
Taking 2 rounds of MDA	64 (17)	122 (18)	65 (18)	180 (18)
Taking 3 rounds of MDA	236 (61)	185 (28)	210 (57)	280 (29)
***Malaria Post access***				
Consultations from the village	575	770	513	1496
Consultations from study participants	515	603	488	889
*P. falciparum* malaria cases from the village	6	89	9	8
*P. vivax* malaria cases from the village	81	139	47	191
Consultations from outside the village	465	390	192	1242
*P. falciparum* malaria cases from outside the village	11	66	4	20
*P. vivax* malaria cases from outside the village	52	67	11	277
Total number of weeks of MP inactivity	6	17	5	4
Mean consultations per 100/active week	3.2	2.0	2.0	3.8

**‡**values presented are averages over 10 census occasions during surveys in the village.

### Mass antimalarial drug administration (MDA)

**Figure 1.**
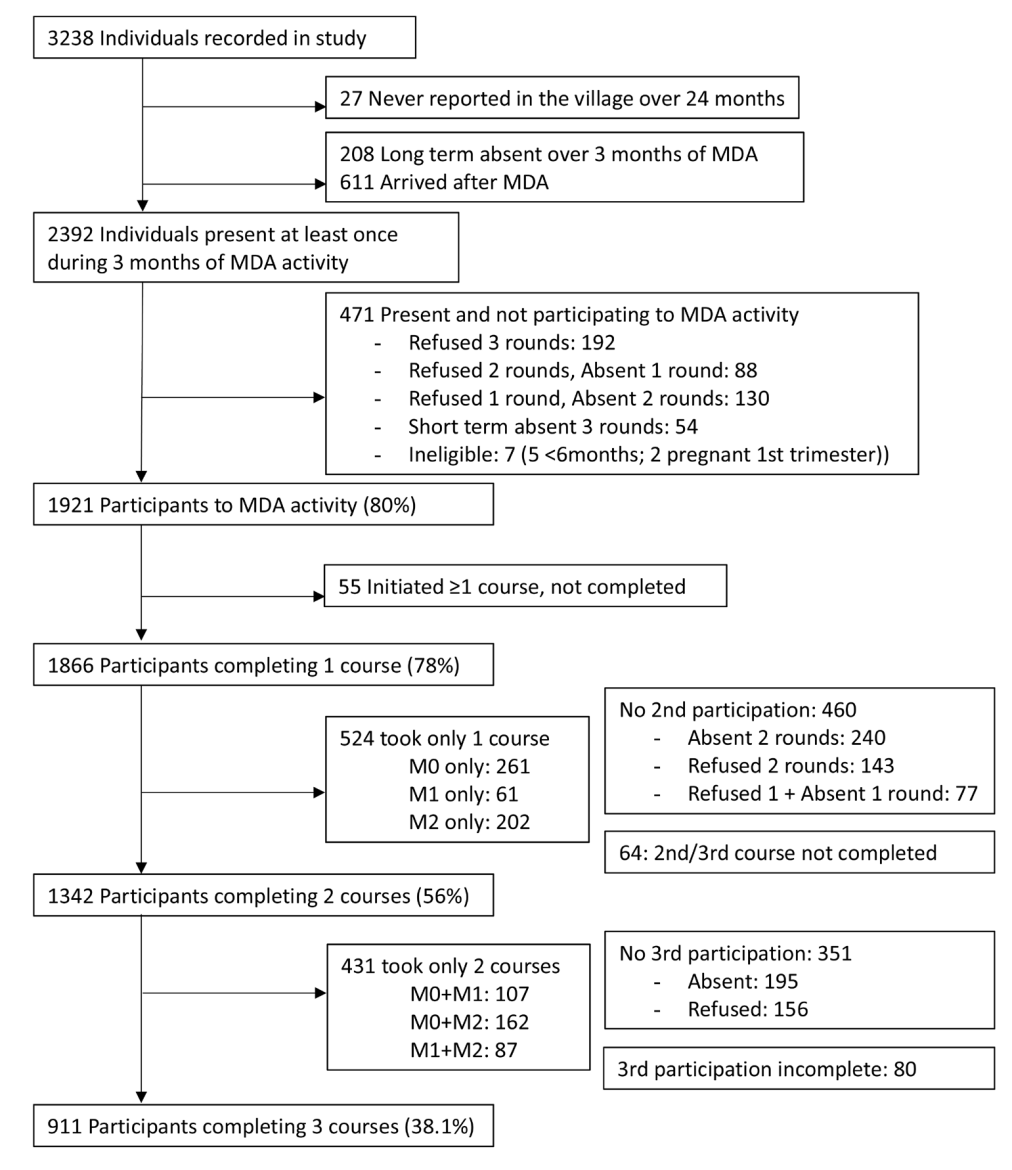
Flow chart of the participation in antimalarial MDA in the four villages.

***MDA coverage***. The target population was 2392 residents of the four villages at the time of MDA. Overall 78% (1866/2392) received at least one 3–day course of DP and 38% (911/2392) received the full course of three treatments. Coverage was higher in the two small villages compared to the two larger villages, for uptake of at least one treatment (A1–KNH: 93%; 360/386 and B1–TPN: 92%; 339/367 compared to A2-TOT: 74%; 487/662 and B2-HKT: 70%; 680/977) and uptake of three treatments (A1–KNH: 61%; 236/386 and B1–TPN: 57%; 210/367 compared to A2-TOT: 28%; 185/662 and B2-HKT:29%; 280/977) ( Table 1; Figure 1).

***Safety and tolerability of MDA***. MDA was well accepted and well tolerated. In 3719 of 3931 (94.6%) complete structured interviews, no AE were reported. Reported AE were in majority dizziness (N=192) and pruritus (N=17). There were three reports of black urine. One G6PD-deficient male reported black urine before primaquine and one G6PD-normal male reported black urine 48h after primaquine. Neither were anaemic and both remained well. The third case, a G6PD heterozygote woman with normal G6PD activity, became anaemic but concomitant HIV and TB were considered contributory, as reported previously ^26^. The percentage of participants reporting AE declined after each MDA round from 8.2% (114/1394) to 3.7% (49/1336). Among the 200 patients who presented at the mobile clinic over the 3-month MDA period, all AE were mild or moderate and most (188/200, 94%) were rated as unrelated or unlikely to be related to treatment ( Table 2). During the 24 months of the study, there were 23 reported serious AE and 15 deaths; none were considered drug related ( Supplementary File 1: Table S2). Haemoglobin decreases in G6PD-deficient individuals were minimal as already reported ^26^.

**Table 2.** Safety of mass drug administration (MDA) intervention in the participant population: severity and relation to drugs of adverse events (AE) investigated at the mobile clinic set up during the three months of MDA (M0–M3 or M9–M12). Severe AEs over the 24-month follow-up are detailed in Supplementary File 1: Table S2.

	Relationship to drug					
Severity	Not related	Unlikely related	Possibly related	Probably related	Highly likely related	Total
**Mild**	134 Infection (71) Weakness (39) Dizziness (4) Allergy (1) Other (19)	49 Infection (5) Weakness (19) Dizziness (23) Allergy (1) Other (1)	7 Infection (2) Weakness (3) Dizziness (1) Allergy (1)	0	1 Allergy (1)	191
**Moderate**	5 Infection (4) Other (1)	0	4 Weakness (2) Allergy (2)	0	0	9
**Severe**	0	0	0	0	0	0
**Total**	139	49	11	0	1	200

### Malaria prevalence

There was a significant difference in baseline *P. falciparum* prevalence between intervention and control villages (18.7% (150/801) versus 6.8% (58/848); p<0.0001).

The majority of malaria infections detected by uPCR during the cross-sectional surveys were asymptomatic (86%; 1769/2059) and 70% were not detected by microscopy or RDT (1432/2059) ( Supplementary File 1: Table S3). Overall, of 217 individuals positive for *P. falciparum*, 17 (8%) were still positive in the following survey 3 months later, three were positive after 6 months, and one after 9 months. DP was highly efficacious; no participant receiving at least one complete 3-day DP course was positive for *P. falciparum* in the survey following MDA (M3 or M12), irrespective of previous infection status ( Supplementary File 1: Table S4 and Table S5). During the follow-up (excluding MDA intervention period), 69% (707/1018) of newcomers to the villages were included in the first survey after their arrival, of whom 1.6% (11/707) were positive for *P. falciparum.*

### Efficacy of MDA

***Pilot trial endpoints: MDA+MP versus MP only comparison***

**Prevalence.** At M3 the prevalence of *P. falciparum* infection had fallen to 0.4% (2/552; 95% CI 0.04 to 1.3), in intervention villages A1-KNH and A2-TOT, and 2.7% (22/812; 1.7 to 4.1) in non-intervention control villages, B1-TPN and B2-HKT (p=0.001). Comparing M0 to M3, prevalence decreased by 98% in MDA villages compared to a 57% decrease in control villages. At M9, the difference in prevalence was no longer significant: 2.0% (11/564; 1.0-3.5) in MDA villages, and 0.9% (9/959; 0.04-1.8) in control villages (p=0.10). Comparing M0 to M9, MDA villages exhibited an 88% decrease in prevalence, compared to an 85% decrease in control villages.

**Incidence.** Between M0 and M9, falciparum malaria incidence was 1.0 case/1000 person-months (95%CI=0.4-1.9) in MDA villages (8/8352) compared with 1.3 case/1000 person-months (0.6-2.5) in control villages (10/7469, p=0.49). *P. vivax* malaria incidence was 8.6 cases/1000 person-months (6.8-10.9) in MDA villages (72/8352), compared with 5.6 cases/1000 person-months (4.1-7.6) in control villages (42/7469, p=0.03).

***Before versus after MDA comparison***

**Figure 2.**
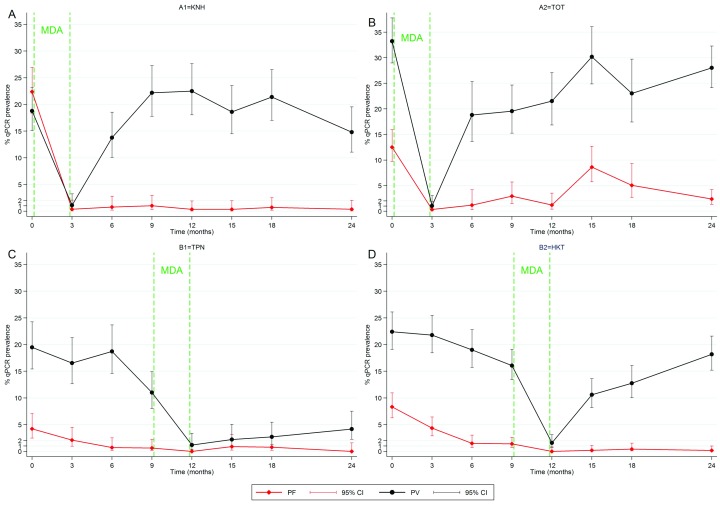
Prevalence of *P. falciparum* and *P. vivax* carriage over 24 months measured by uPCR in quarterly cross sectional surveys conducted in the four study villages. A steep decrease in prevalence immediately followed MDA in all 4 villages. The reduction in *P. falciparum* prevalence was sustained in 3 of 4 villages stabilizing to a <1% prevalence after MDA. In contrast *P. vivax* prevalence rebounded in 3 of 4 villages. In village A2-TOT, *P. falciparum* prevalence increased at M15 and M18, mostly from clinical cases occurring while vector densities and thus transmission was increased ( Table 5). A decrease in *P. falciparum* prevalence was observed in the control period (M0–M9) in villages B1 and B2, possibly attributable to the effect of early diagnosis and treatment provided by MP.

***P. falciparum* infection**. The impact of MDA on *P. falciparum* carriage was different in the two intervention villages. In A1–KNH, there was a rapid >95% reduction in *P. falciparum* prevalence, which was sustained from M3 to M24 ( Figure 2). In contrast, in village A2–TOT, prevalence fell between M0 and M3, to reach a peak at M15 during rainy season ( Figure 2). In this village, prevalence was maintained around 5% among individuals who did not take MDA, from M3 to M24 ( Supplementary File 1: Figure S4). Prevalence in the control villages decreased gradually over the control period, dropping at 0% at M12 immediately after MDA, and remained below after MDA ( Figure 2).

Adjusting for age group, sex, village and season, MDA was associated with an overall 12-fold decrease in odds of *P. falciparum* carriage over the full observation period (21 month for intervention and 15 months for controls) compared to baseline (OR [95%CI]=0.08 [0.05–0.11]) ( Table 3). The control period before MDA was also associated with a significant decrease in odds of *P. falciparum* carriage (0.4 [0.3–0.7]) ( Table 3). This translated into a significant 5-fold odds reduction after MDA compared to control period (0.2 [0.1–0.3]).

**Table 3.** Multivariable analysis of factors associated with *Plasmodium falciparum* carriage during uPCR surveys over the 24-month study period and quantification of the impact of mass drug administration (MDA). Two specifications of the MDA impact are presented showing overall impact by period (model 1) or including temporal trends (model 2). The impact of individual participation is presented in Supplementary File 1: Table S7 (see also Supplementary File 1: Table S6 for univariable analysis). The direct comparison between control and intervention adjusting for baseline *P. falciparum* infection in presented in Supplementary File 1: Table S8 and Supplementary File 1: Table S9. Baseline refers to M0 survey at the beginning of the study. Control period refers to the period between M0 and M9 in the two control villages after implementation of Malaria Post and LLIN distribution at M0. After MDA refers to the period where MDA had been conducted in addition to implementation of Malaria Post and LLIN distribution (M3 to M24 in intervention villages, M12 to M24 in control villages).

Variable	Categories		Adjusted OR	95%CI	p–value
**Age**	≤10 years		1	Reference	<0.0001
	>10 years		2.8	1.9–4.1	
**Sex**	Female		1	Reference	<0.0001
	Male		2.3	1.7–3.2	
**Village**	B2–HKT		1	Reference	<0.0001
	B1–TPN		0.8	0.4–1.4	
	A2–TOT		4.0	2.4–6.5	
	A1–KNH		2.8	1.9–4.0	
**Season**	Cold		1	Reference	<0.0001
	Hot		1.0	0.6–1.6	
	Wet		2.5	1.6–4.1	
**Model 1. Study period**	Baseline (M0 survey)		1	Reference	<0.0001
	Control period		0.4	0.3–0.7	
	After MDA period		0.08	0.05–0.11	
**Model 2. Interaction between** **study period and time**	Study period	Baseline (M0 survey)	1	Reference	<0.0001
		Control period	0.4	0.2–0.8	
		After MDA period	0.03	0.01–0.05	
	Time (for each additional month)	during control period	1.03	0.91–1.16	
		during period after MDA	1.08	1.03–1.12	

Compared to baseline, the decrease in odds of *P. falciparum* carriage was 30-fold immediately after MDA (0.03 [0.02–0.06]). Each additional month after MDA was associated with a minimal increase in odds of *P. falciparum* carriage (1.08 [1.03–1.12] for one additional month). For example, 12 months after MDA, a 15-fold reduction persisted (0.06 [0.04–0.09]). Similar results were obtained when estimating odds for each quarter instead of assuming a monthly linear trend ( Supplementary File 1: Figure S5).

During the pre-MDA control period, individuals with baseline *P. falciparum* infections had a significantly increased odds of subsequent *P. falciparum* carriage compared to baseline uninfected individuals (17.2 [6.5–45.6]) ( Supplementary File 1: Table S8). Irrespective of their baseline infection status, individuals taking one or two rounds and individuals completing the three rounds of MDA had a significantly decreased odds of being infected with *P. falciparum* compared to individuals in the control period (0.2 [0.1–0.6] and 0.1 [0.03–0.4] respectively). In contrast, individuals who did not receive MDA in villages allocated to it had no decrease in the odds of *P. falciparum* carriage (0.4 [0.1–1.9]) compared to the control period ( Supplementary File 1: Table S8).

**P. falciparum clinical malaria**. A total of 113 *P. falciparum* symptomatic cases were detected among study participants, of which 91 were detected passively by the MP and 22 were detected during surveys. An additional 19 cases were detected among non-participants (individuals who reported living in the village but were not recorded in the study, e.g. visitors).

**Figure 3.**
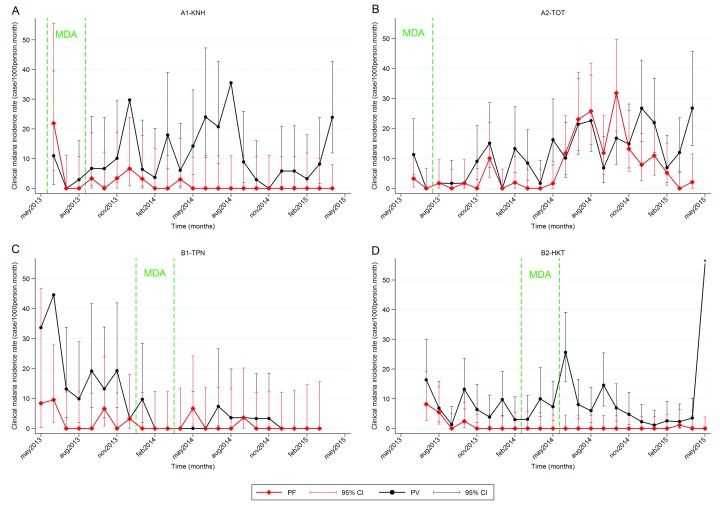
Changes in the incidence of *P. falciparum* and *P. vivax* clinical cases. Clinical falciparum malaria cases persisted up to 9 months in villages achieving stable <1% *P. falciparum* prevalence after MDA (A1, B1 and B2), before zero incidence was achieved. In village A2, where the intervention with MDA was not considered successful, a significant increase in incidence was observed during the rainy and cold seasons from July 2014 to January 2015.* *P. vivax* incidence rate in B2-HKT for June 2015 was 100 cases/1000 person-month (95%CI=81-122).

In 3 out of 4 villages, the combined intervention of early diagnosis and treatment through the MP and MDA resulted in a dramatic and sustained decrease in the incidence of *P. falciparum* clinical cases, which was close to zero after 12 months ( Figure 3). Most of the clinical episodes (75/113, 66%) occurred during a 7–month period in village A2–TOT between July 2014 and January 2015 (M14 to M20), corresponding to the rainy and following cool seasons.

**Table 4.** Detection of molecular markers of antimalarial resistance in *Plasmodium falciparu* *m* positive samples according to period before and after mass drug administration (MDA).

	Before MDA	After MDA	Overall
PF positive samples by uPCR	191	56	247
Sample with PfK13 result	77 (40.3%)	30 (53.6%)	107 (43.3%)
Samples with PfK13 mutation	66	17	83
PfK13 Mutation prevalence [95% CI]	85.6% [75.9–92.6]	56.7% [37.4–74.5]	77.6% [68.5–85.1]
Sample with Piperaquine resistance result	53 (27.7%)	16 (28.6%)	69 (27.9%)
Samples with Piperaquine resistance marker	0	0	0
Piperaquine resistance prevalence [95% CI]	0% [0–6.7]	0% [0–20.6]	0% [0–5.2]

***P. vivax infection and clinical malaria***. In 3 out of 4 villages, *P. vivax* prevalence was reduced only transiently by MDA ( Figure 2). Even when MDA participation was high, *P. vivax* prevalence rebounded quickly to reach initial levels within 3 to 6 months. In village B1–TPN, *P. vivax* prevalence did not rebound as rapidly. *P. vivax* symptomatic malaria incidence was unaffected beyond the 3-month MDA period in three villages, with seasonal peaks of clinical cases during transmission seasons both before and after MDA ( Figure 3). In village B1–TPN, the incidence did not return to pre-MDA levels.

### Prevalence of molecular markers of antimalarial drug resistance

Data on the artemisinin resistance marker *Pfkelch13* (PfK13) was available for 107 of 247 detected *P. falciparum* infections. The prevalence of PfK13 mutations was 77.6% (83/107) ( Table 4). The most frequent mutations were C580Y (37.4%; 31/83) and G538V (24.1%; 20/83). The prevalence of PfK13 mutants was 85.6% (95%CI=75.9–92.6) before, and 56.7% (37.4–74.5) after MDA.

No plasmepsin 2 amplification (the molecular marker of piperaquine resistance) was found in the 69 samples that could be analysed (53 before MDA and 16 after) ( Table 4).

### Entomological findings

The main anopheles vectors in the villages belonged to the Minimus Complex (41% of total anopheles), Maculatus Group (11%) and Dirus Complex (1%). Overall, the SI of primary malaria vectors was 2.2/1000 for *P. vivax* and 0.4/1000 for *P. falciparum* ( Table 5). During the period before MDA, the HBR was between 200 and 300 bites per human per month in all four villages. After MDA, the HBR was unchanged in A1-KNH and B2-HKT villages, decreased in B1-TPN, and increased in A2-TOT. In spite of these variations in abundance of vectors, SI and EIR decreased after MDA in A2-TOT. Before MDA, *P. falciparum* SI was 0.9/1000 (95%CI=0.5-1.6) compared to 0.2 (95%CI=0.05-0.4) after (p=0.0001). Likewise, before MDA, *P. falciparum* EIR was 0.25 infective bites/human/month (95%CI=0.13-0.43) compared to 0.06 (95%CI=0.02-0.15) after (p=0.005).

**Table 5.** Human biting rate (HBR, bites/person/month), sporozoite index (SI, positive/1000) and entomological inoculation rate (EIR, infective bites/person/month) for primary malaria vectors ( *Anopheles minimus*, *An. maculatu* *s* and *An. dirus*) by study village according to period before and after mass drug administration (MDA) intervention.

		Before MDA ‡			After MDA			All follow-up		
		n/N	index	95%CI	n/N	index	95%CI	n/N	index	95%CI
**A1-KNH**	Human.nights	200			850			1050		
	HBR	1615/6.67 *	242	[231-254]	7803/28.33 *	275	[269-282]	9418/35.00 *	269	[264-275]
	Pf-SI	3/1574 †	1.9	[0.4-5.6]	1/7307 †	0.1	[0.004-0.8]	4/8881 †	0.5	[0.1-1.2]
	Pf-EIR		0.46	[0.01-1.36]		0.04	[0.001-0.21]		0.12	[0.03-0.31]
**A2-TOT**	Human.nights	150			900			1050		
	HBR	1776/5.00 *	355	[339-372]	22438/30.00 *	748	[738-758]	24214/35.00 *	692	[683-701]
	Pf-SI	3/1729 †	1.7	[0.4-5.1]	3/20742 †	0.1	[0.03-0.4]	6/22471 †	0.3	[0.1-0.6]
	Pf-EIR		0.61	[0.13-1.88]		0.10	[0.02-0.30]		0.17	[0.07-0.39]
**B1-TPN**	Human.nights	600			400			1000		
	HBR	4500/20.00 *	225	[218-232]	1419/13.33 *	106	[101-112]	5919/33.33 *	178	[173-182]
	Pf-SI	2/4265 †	0.5	[0.1-1.7]	1/1271 †	0.8	[0.02-4.4]	3/5536 †	0.5	[0.1-0.6]
	Pf-EIR		0.12	[0.01-0.38]		0.08	[0.002-0.46]		0.11	[0.02-0.28]
**B2-HKT**	Human.nights	670			350			1020		
	HBR	6771/22.33 *	302	[296-310]	3870/11.67 *	332	[321-342]	10641/34.00 *	312	[307-319]
	Pf-SI	5/6632 †	0.8	[0.2-1.8]	0/3809 †	0	[0-1]	5/10441 †	0.5	[0.2-1.1]
	Pf-EIR		0.21	[0.07-0.54]		0	[0-0.36]		0.14	[0.05-0.36]
**4 villages**	Human.nights	1620			2500			4120		
	HBR	14662/54.00 *	272	[267-276]	35530/83.33 *	426	[422-431]	50192/137.33 *	365	[362-369]
	Pf-SI	13/14200 †	0.9	[0.5-1.6]	5/33129 †	0.2	[0.05-0.4]	18/47329 †	0.4	[0.2-0.6]
	Pf-EIR		0.25	[0.13-0.43]		0.06	[0.02-0.15]		0.14	[0.08-0.22]

‡ 1 to 2 surveys conducted during MDA intervention are included for each village * n captured/N person.time exposed (human.months) † n positive/N tested

## Discussion

The rapid emergence of artemisinin resistance followed by ACT partner drug resistance in *P. falciparum* parasites in the GMS leaves few options to prevent worsening malaria and spread of resistance westwards, as has occurred before. This preliminary evaluation of the feasibility, safety and effectiveness of MDA in accelerating the elimination of artemisinin-resistant *P. falciparum* in communities with a high prevalence of asymptomatic malaria shows that MDA with DP and a single low dose of primaquine was safe and well accepted ^26^. Most participants did not report any AE, and those who did reported only mild and transient AE which declined in frequency after each successive round. Access to diagnosis and treatment, an essential component of malaria control, was deployed at baseline and had a substantial impact on malaria, which was augmented by MDA. When coverage was high, MDA provided a marked and long-lasting impact on *P. falciparum* malaria prevalence and incidence, but these benefits were transient in one village (A2-TOT). Overall, the odds of *P. falciparum* carriage decreased by >95% immediately after MDA in comparison with baseline and an 80% decrease was maintained for 24 months.

The effect of these interventions on *P. vivax* malaria was substantially less important, as expected. The MDA did not target hypnozoites, so relapses of vivax were not prevented and *P. vivax* incidence and prevalence rose rapidly after the period of post treatment prophylaxis. Because of the relatively high prevalence of G6PD deficiency in this population and the unavailability of rapid testing, radical cure with primaquine was not used in *P. vivax* malaria treatment. Interestingly, in village B1-TPN only, the prevalence of *P. vivax* infection fell and did not rebound.

### Limitations of the study

The main limitation of this study is that, being a pilot investigation, it is underpowered to draw definitive conclusions on the overall benefit of this approach. The comparison between intervention and control is indeed strongly influenced by individual village characteristics: although the four villages had similar malaria prevalence in the pre-survey ( Table 1, Supplementary File 1: Table S1), they were significantly different in the baseline exhaustive survey with a higher prevalence of asymptomatic malaria in the villages receiving MDA first.

A second limitation was that the beneficial effects of MDA could not be evaluated independently from the continuing effects of the malaria post and the LLINs. Their benefits are illustrated in the control villages where there was three-fold reduction in *P. falciparum* prevalence over a nine month observation period until MDA was conducted. As the two control villages had a relatively low baseline *P. falciparum* prevalence, the impact in a higher prevalence setting of instituting a malaria post and distributing LLIN without providing MDA cannot be predicted from these observations. Nevertheless providing MDA in these control villages after nine months reduced prevalence by 100% immediately after MDA, showing an additional effect of MDA to enhanced control measures, as shown recently in Zambia ^15^.

Third, those who refused MDA were less likely to participate in surveys (as in village A2-TOT). This led to underestimation of the true prevalence of malaria, which was close to zero in the general survey population but 5% in the group of survey participants who did not take MDA. Individual participation in MDA was therefore included in the statistical models of impact to take this into account.

### Interpretation

The sustained reduction in *P. falciparum* prevalence in village A1-KNH, where MDA coverage was high, suggests a reduction of malaria transmission. This is supported by finding reductions of similar magnitude in *P. falciparum* SI and EIR, while HBR remained stable. By contrast, in A2–TOT, *P. falciparum* prevalence and incidence increased as vector abundance rose during the rainy and cold seasons of 2014, 14 to 20 months after MDA ( Table 5). In this community broken by years of conflict, participation with MDA was low and the reservoir of sub-microscopic malaria parasite carriage was reduced only partially. The non-MDA participants may have contributed as a reservoir for *P. falciparum*, as indicated by a continuously high prevalence of asymptomatic *P. falciparum* carriage (5%) in this group. In addition, the MP was not functioning properly during the first year, with frequent interruptions of activity and the lowest rate of consultations among the four villages ( Table 1 and Supplementary File 1: Figure S6). These observations illustrate the ‘real-life’ challenges that would confront a programme aiming for rapid elimination, and confirm the well described necessity of ensuring high coverage in any MDA campaign. They also underline the essential importance of providing diagnosis and effective treatment, both immediately after MDA and in an outbreak context as experienced in A2-TOT between M14 and 20. The low prevalence observed at M24 (2%) is likely the result of the adequate treatment of clinical episodes by the MP during the period M12 to M24, contrasting with the dysfunctions observed between M0 and M12.

Importantly this intervention does not appear to have modified the resistance profile of *P. falciparum* in the area. The prevalence of mutations in the artemisinin resistance marker PfK13 did not increase and there was no indication that piperaquine resistance was selected, although numbers were small. No markers of piperaquine resistance were found either in >200 samples from clinics covering the same area and period, where DP was used for routine treatment of clinical cases (Miotto O, unpublished data).

## Conclusion

This pilot study shows that MDA with three rounds of a treatment course of DP and a single low dose of primaquine at one month interval is well tolerated, safe and feasible once the community has been successfully engaged in participation to activities and ownership of the program. It provides evidence that in a remote malaria endemic area, following implementation and support of a functioning MP, and distribution of LLIN, MDA eliminates the sub-microscopic parasites in those who take it. If a sufficiently high proportion of the community participates and if early diagnosis and treatment of clinical episodes remains available, this has a long lasting effect on falciparum malaria reservoir. By contrast, *P. vivax* returns rapidly, presumably because of relapse, which occurs frequently in the GMS. These preliminary findings need confirmation and exploration in further studies. Scaling up to provide MDA to larger populations will introduce new challenges of feasibility, but may improve overall effects as human migration from untreated areas becomes less likely. From a community perspective, elimination of malaria means elimination of all malaria, and to achieve that rapidly will require targeting the vivax hypnozoite reservoir.

## Data availability

The data referenced by this article are under copyright with the following copyright statement: Copyright: © 2017 Landier J et al.

The data is available upon request to the Mahidol Oxford Tropical Medicine Research Unit Data Access Committee ( Supplementary File 2; http://www.tropmedres.ac/data-sharing) and following the Mahidol Oxford Tropical Medicine Research Unit data access policy ( http://www.tropmedres.ac/_asset/file/data-sharing-policy-v1-0.pdf).

## Supplementary material

Supplementary File 1: Supplementary results.

Supplementary File 2: Request form for Mahidol Oxford Tropical Medicine Research Unit Data Access Committee.
