# Supplementary material for: Safety and effectiveness of mass drug administration to accelerate elimination of artemisinin-resistant falciparum malaria: A pilot trial in four villages of Eastern Myanmar
Source: Wellcome Open Res. 2017 Sep 6;2:81. [Version 1] doi: 10.12688/wellcomeopenres.12240.1 (PMC5635445; doi:10.12688/wellcomeopenres.12240.1)
Supplement: Supplementary file 1 [file wellcomeopenres-2-13252-s0000.tgz › ee3d80ca-9f4e-4013-8dde-5b737c90b109.docx]

**Supplementary File 1: Safety and effectiveness of mass drug administration to accelerate elimination of artemisinin-resistant falciparum malaria: A pilot trial in four villages of Eastern Myanmar**

***Supplementary results***

1. **Pre-study survey results**

Based on the pre-survey results, the prevalence of *P. falciparum* infection was 10% in average across the 4 villages with no significant difference between intervention and control villages (12.0% (11/92) and 8.8% (9/102) respectively, p=0.47).

**Table S1: Pre-study screening survey results. Prevalence of malaria as measured by ultrasensitive qPCR in 12 villages from Eastern Myanmar along the border with Thailand.** PF, *Plasmodium falciparum*; PV, *P. vivax.*

| Village | qPCR results | | | | | Total samples | PF prevalence (%) | Malaria prevalence (%) | % PF among positive | randomized |
| --- | --- | --- | --- | --- | --- | --- | --- | --- | --- | --- |
|  | Neg | PF | PF+PV | PV | Plasmodium spp. |  |  |  |  |  |
| HKT | 28 | 7 | 0 | 9 | 4 | 48 | 14.6 | 41.7 | 35.0 | B2 |
| KG | 40 | 0 | 0 | 5 | 4 | 49 | 0.0 | 18.4 | 0.0 | no |
| KNH | 29 | 6 | 2 | 11 | 3 | 51 | 15.7 | 43.1 | 36.4 | A1 |
| LG | 42 | 0 | 0 | 5 | 3 | 50 | 0.0 | 16.0 | 0.0 | no |
| LH | 50 | 1 | 1 | 2 | 1 | 55 | 3.6 | 9.1 | 40.0 | no |
| LP | 56 | 1 | 0 | 3 | 0 | 60 | 1.7 | 6.7 | 25.0 | no |
| MPK | 35 | 1 | 0 | 13 | 1 | 50 | 2.0 | 30.0 | 6.7 | no |
| TOT | 24 | 3 | 0 | 10 | 4 | 41 | 7.3 | 41.5 | 17.6 | A2 |
| TPN | 37 | 1 | 1 | 4 | 11 | 54 | 3.7 | 31.5 | 11.8 | B1 |
| WK | 41 | 0 | 0 | 4 | 5 | 50 | 0.0 | 18.0 | 0.0 | no |
| WT | 47 | 0 | 0 | 4 | 0 | 51 | 0.0 | 7.8 | 0.0 | no |
| YK | 44 | 1 | 1 | 3 | 7 | 56 | 3.6 | 21.4 | 16.7 | no |

1. **Location of study sites**

**
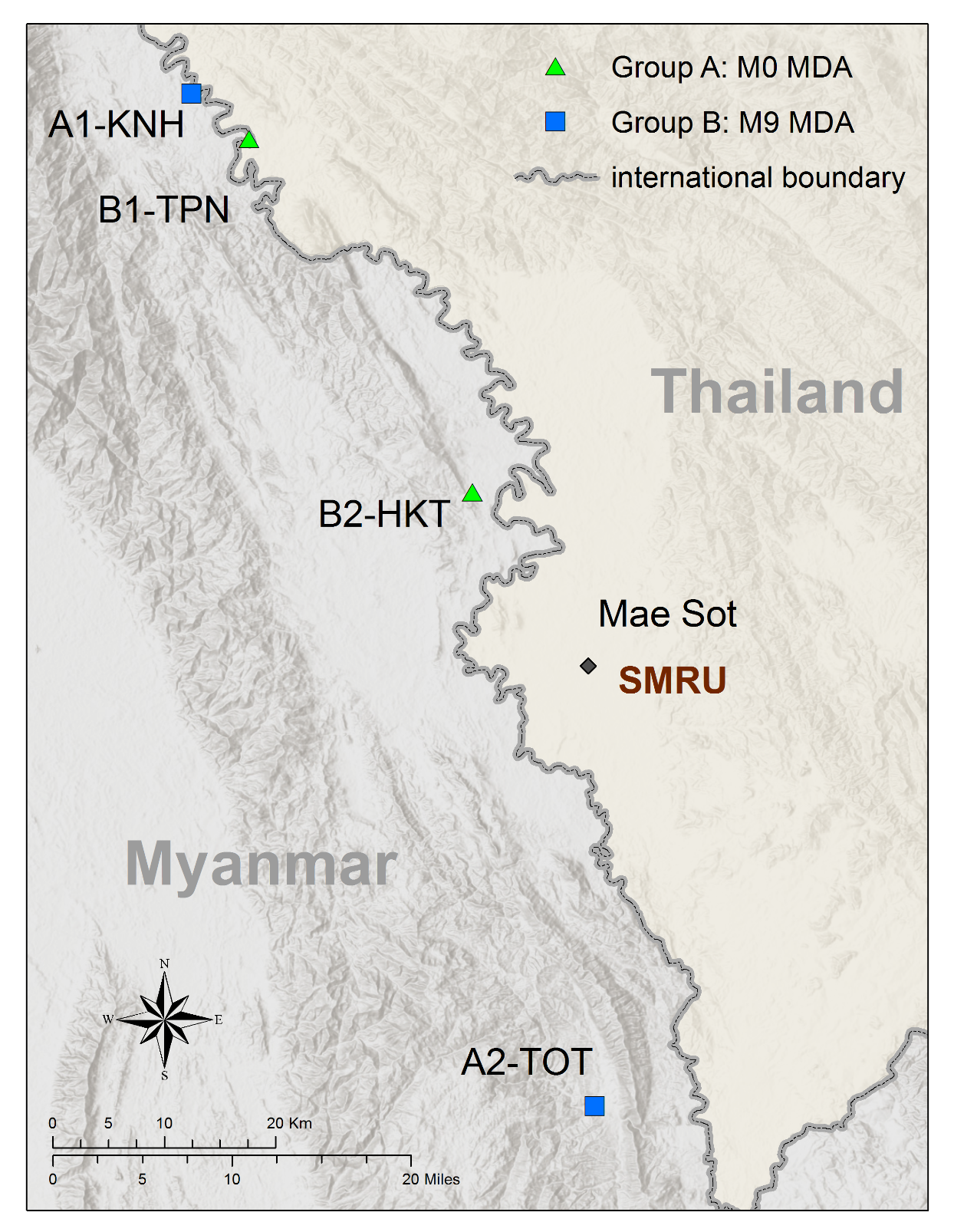
**

**Figure S1: Location of the study villages in Eastern Kayin (Karen) state, Myanmar, close to the border between Myanmar and Thailand.** The two closest villages (A1-KNH and B1-TPN) were only separated by 6 km in a straight line but actual paths from one village to the other were longer, especially during the rainy season. There were no patient from one village recorded in the other village malaria post (MP), out of 1043 and 786 consultations, respectively; and the M0 prevalence was markedly different between the two villages. However, patients from neighbouring villages further inland could be found in either MP.

1. **Demography**


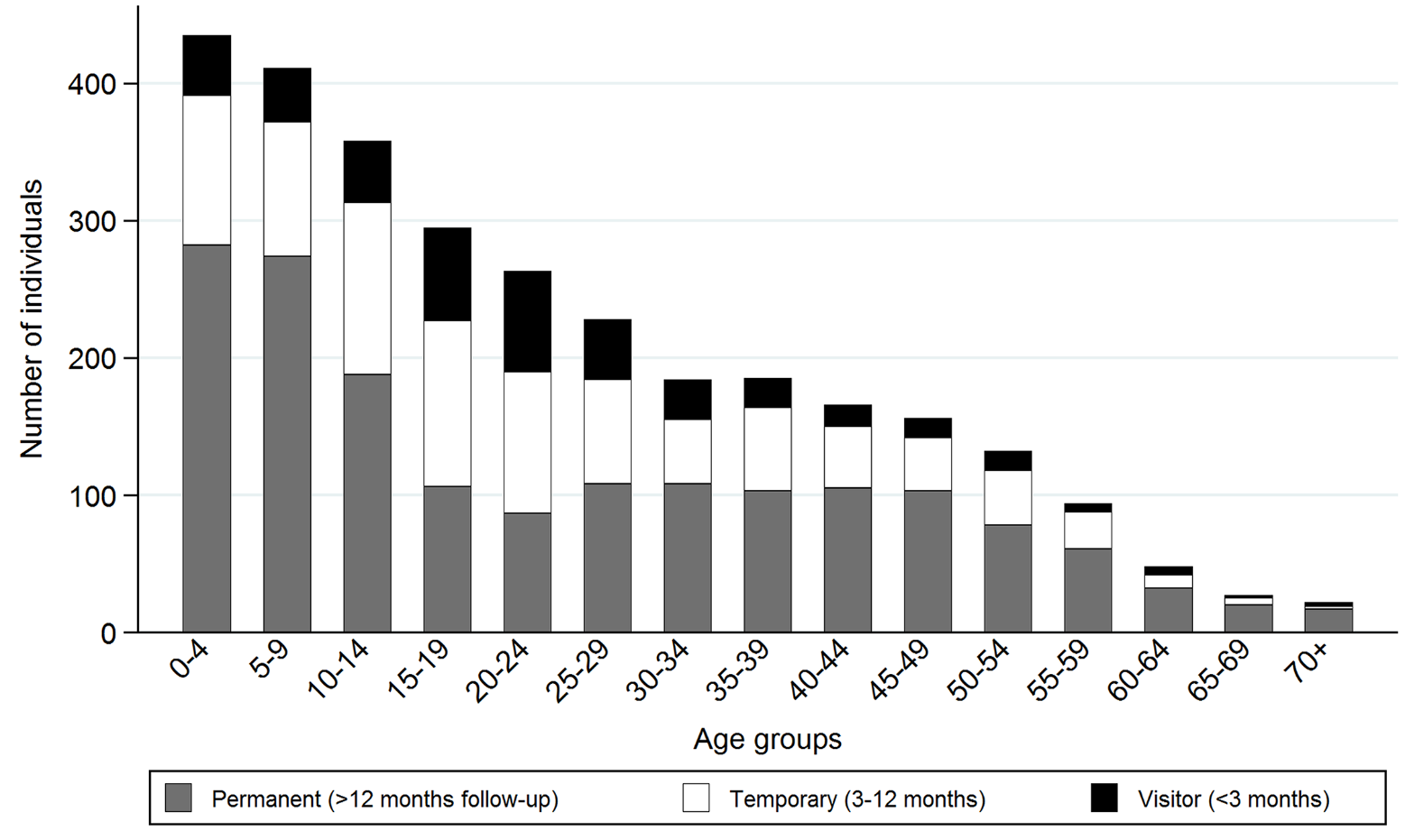


**Figure S2: Age distribution of the study population according to cumulative follow-up time.** The age structure of the study population shows movement patterns which are typical from rural areas in the region where the study was located. A majority of individuals between 15 and 35 years accumulated less than 12 months of follow-up. These individuals leave the village for long periods of time to attend boarding schools or conduct seasonal works.


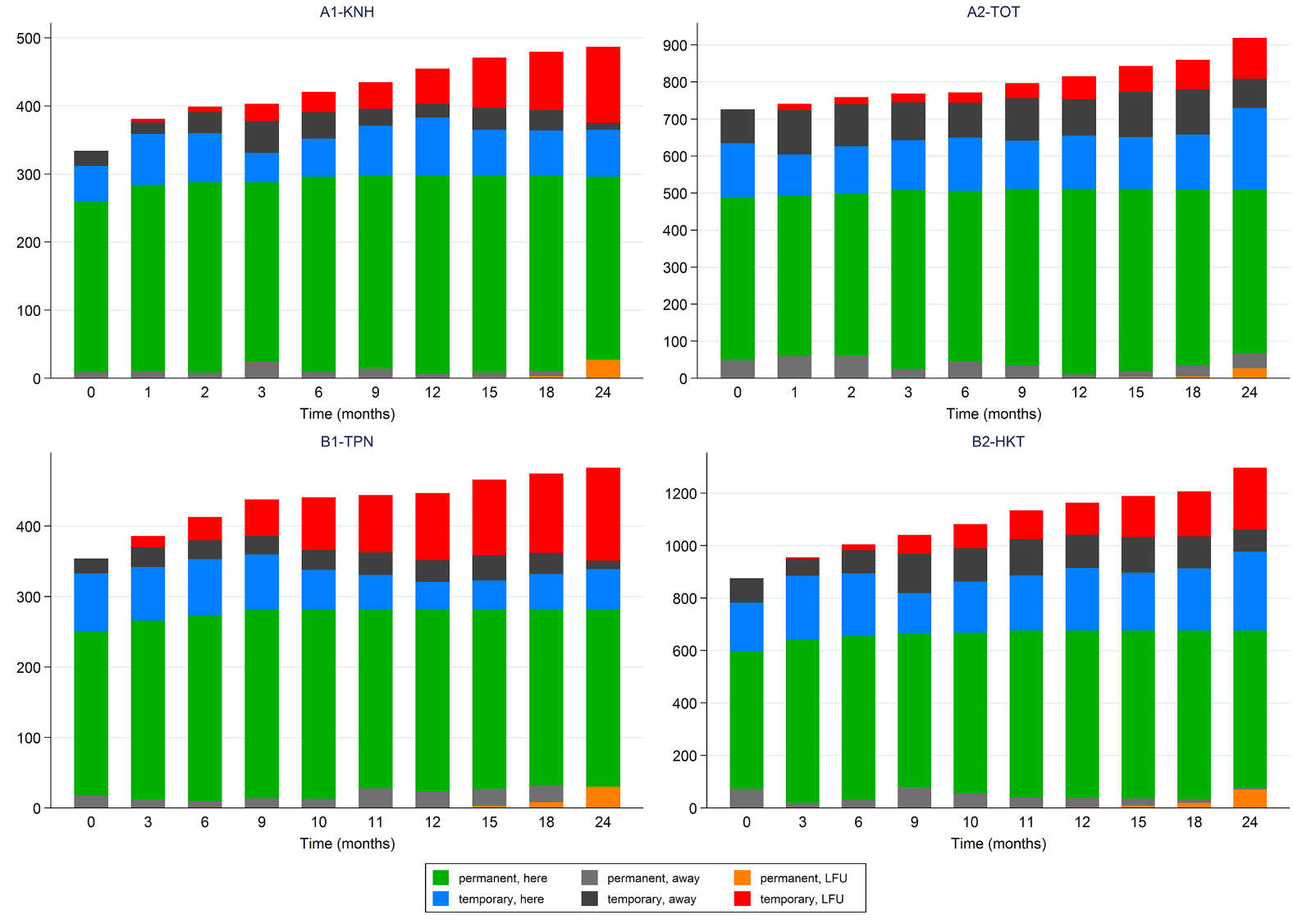


**Figure S3: Quarterly population follow-up in the four study villages.** Permanent population represents individuals who accumulated over 12 months of follow-up during the 24 months of study. Temporary population represent individuals with less than 12 months of follow-up. Status is categorized as in the village (here), away (if coming back) or lost to follow-up (LFU).

1. **Safety**

**Table S2: Detailed record of all Severe Adverse Events during the 24 months of follow-up**

| **Date of onset** | **Diagnosis** | **Severity category** | **Date of death** | **Site** | **Date of admission** | **Date of discharge** |
| --- | --- | --- | --- | --- | --- | --- |
| 14/07/2013 | Septic abortion (3 months) | life threatening | NA | Refugee camp clinic | 14/07/2013 | 19/07/2013 |
| 29/07/2013 | Alcoholic stroke | life threatening | NA | Refugee camp clinic | 29/07/2013 | 08/08/2013 |
| 16/08/2013 | Melioidosis | died | 11/09/2013 | Home | NA | NA |
| 09/09/2013 | Diarrhea | life threatening | NA | Thai Hospital | 10/09/2013 | 12/09/2013 |
| 15/10/2013 | Heart failure | died | 22/10/2013 | Home | NA | NA |
| 17/10/2013 | Rabies | died | 20/10/2013 | Home | NA | NA |
| 26/10/2013 | Tuberculosis | life threatening |  | Myanmar Hospital | 27/10/2013 | 28/03/2014 |
| 22/11/2013 | Stroke | died | 02/12/2013 | Home | NA | NA |
| 24/11/2013 | Septic abortion, Anemia | life threatening | NA | Migrant clinic in Thailand | 24/11/2013 | 30/11/2013 |
| 26/12/2013 | Tuberculosis | life threatening | NA | TB migrant clinic | 26/12/2013 | 26/02/2014 |
| 20/01/2014 | Severe acute abdomen | died | 20/01/2014 | Home | NA | NA |
| 24/02/2014 | Respiratory failure, hypertension | died | 25/02/2014 | Home | NA | NA |
| 30/03/2014 | Myocardial infected | died | 02/04/2014 | Home | NA | NA |
| 31/03/2014 | Motorbike accident/Asthma | life threatening | NA | Myanmar Hospital | 31/03/2014 | 02/04/2014 |
| 01/04/2014 | Tuberculosis | life threatening | NA | Myanmar Hospital | NA | NA |
| 01/04/2014 | Road traffic accident | died | 01/04/02014 | Hospital? | NA | NA |
| 30/04/2014 | Still birth (EGA 35+3) | life threatening | NA | Home | NA | NA |
| 15/05/2014 | Face Burning/ Accident | life threatening | NA | Home | NA | NA |
| 17/05/2014 | Severe malnutrition, Sepsis | died | 24/05/2014 | Migrant clinic in Thailand | 17/05/2014 | 24/05/2014 |
| 17/05/2014 | Tuberculosis/HIV | life threatening | NA | TB migrant clinic | 17/05/2014 | 17/07/2014 |
| 27/05/2014 | Complete abortion | life threatening | NA | Home | NA | NA |
| 02/06/2014 | Septic abortion | life threatening | NA | Migrant clinic in Thailand | 02/06/2014 | 09/06/2014 |
| 22/07/2014 | Diphtheria | died | 26/07/2014 | Thai Hospital | 25/07/2014 | 26/07/2014 |
| 28/07/2014 | Liver Cancer | died | 19/11/2014 | Home | NA | NA |
| 20/08/2014 | Gastritis, hypertension | life threatening | NA | Thai Hospital | 23/08/2014 | 30/08/2014 |
| 10/09/2014 | Appendicitis | life threatening | NA | Thai Hospital | 10/09/2014 | 13/09/2014 |
| 28/09/2014 | Gun accident | prolonged hospitalization | NA | Thai Hospital | 28/09/2014 | 30/10/2014 |
| 08/10/2014 | Urinary tract infection | life threatening | NA | Refugee camp clinic | 18/09/2014 | 30/09/2014 |
| 12/10/2014 | Falciparum+vivax malaria, anemia | life threatening | NA | Migrant clinic in Thailand | 14/10/2014 | 19/10/2014 |
| 19/10/2014 | Acute abdomen pain | died | 21/10/2014 | Migrant clinic in Thailand | 19/10/2014 | 21/10/2014 |
| 28/10/2014 | Stroke | life threatening | NA | Thai Hospital | 28/10/2014 | 04/11/2014 |
| 15/11/2014 | Accident by car | died | 15/11/2014 | Home | NA | NA |
| 23/01/2015 | Severe anemia +Gastritis | life threatening | NA | Thai Hospital | 23/01/2015 | 28/01/2015 |
| 01/02/2015 | Motorbike accident | life threatening | NA | Myanmar Hospital | 01/02/2015 | 09/02/2015 |
| 07/02/2015 | Hypertension, Vivax malaria | life threatening | NA | Myanmar Hospital | 07/02/2015 | 09/02/2015 |
| 13/04/2015 | Hypertension+Diabetes | died | 16/04/2015 | Home | NA | NA |
| 22/04/2015 | Hyperthyroidism +Anemia | died | 14/06/2015 | Village clinic | ND | ND |
| 06/05/2015 | Accident Wound | life threatening | NA | Thai Hospital | 06/05/2015 | 19/05/2015 |

1. **Malaria prevalence**

***5.1 Malaria prevalence by group of MDA uptake in intervention villages***


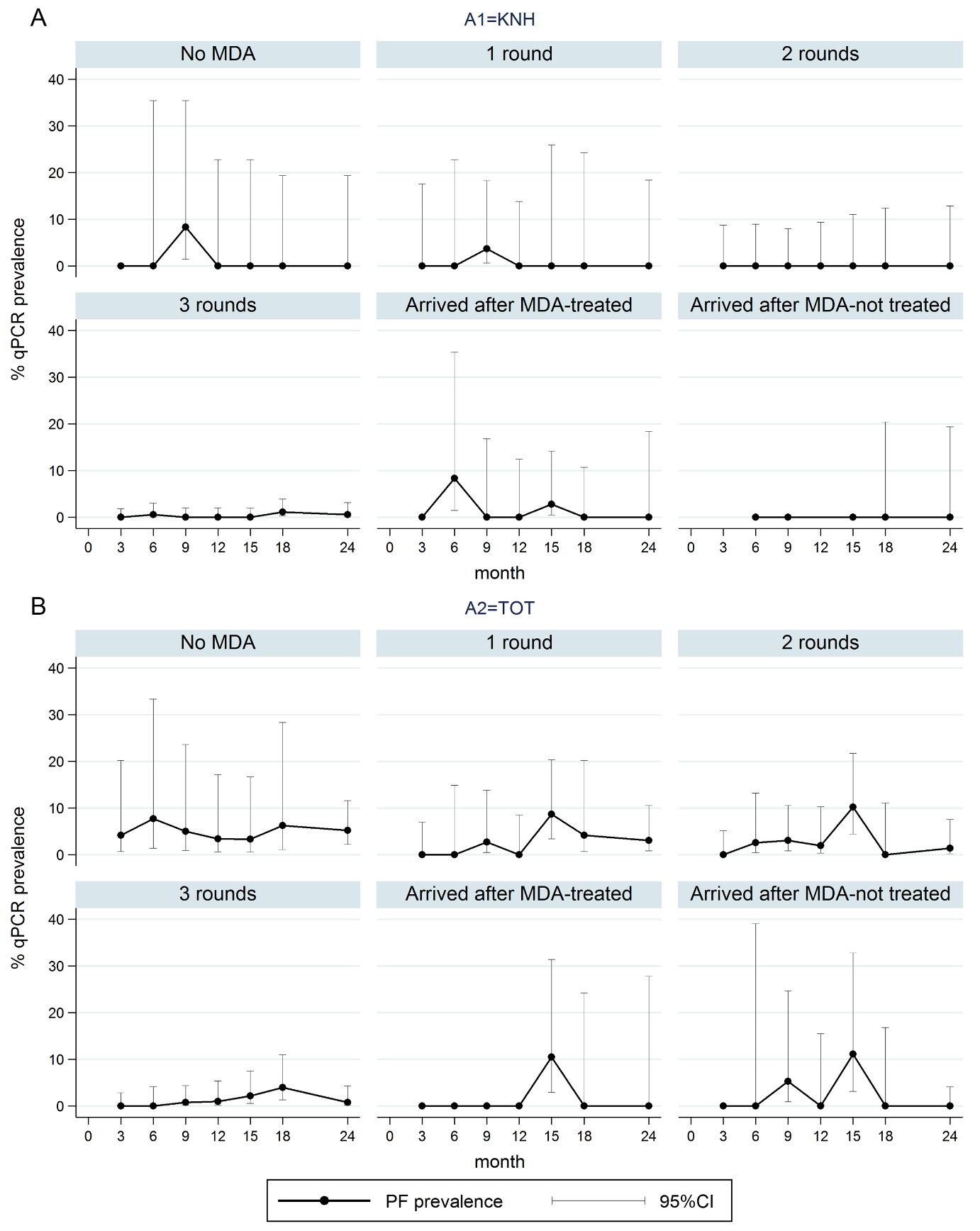


**Figure S4: Evolution of *P. falciparum* prevalence after MDA in the 2 intervention villages by groups of MDA uptake, as measured by uPCR.**

***5.2 Detailed survey results***

**Table S3: Detailed results from quarterly surveys conducted in the four villages and analysed by uPCR.** Number (N) and prevalence (%) of *P. falciparum* (PF), *P. vivax* (PV) and *Plasmodium* spp. (P.spp) infections, as well as total village population during the survey and total number of samples. Of note, immigrants during MDA activity (M1, M2 or M10, M11) were sampled prior to first MDA uptake and were included in the calculation of M0 or M9 prevalence. * ratio not defined (PF incidence=0 and PV incidence=0)

| **Village** | **Month** | **Population present in village** | **N Samples** | **% population included in survey** | **Ratio PF/PV in clinical cases** | | **Prevalence all PF (%)** | **Prevalence all PV (%)** |
| --- | --- | --- | --- | --- | --- | --- | --- | --- |
| A1-KNH | 0 | 385 | 362 | **94%** | | 1.0 | **22.4** | **18.8** |
| A1-KNH | 3 | 307 | 268 | **87%** | | 1.0 | **0.4** | **1.1** |
| A1-KNH | 6 | 344 | 254 | **74%** | | 0.1 | **0.8** | **13.8** |
| A1-KNH | 9 | 357 | 293 | **82%** | | 0.3 | **1.0** | **22.2** |
| A1-KNH | 12 | 377 | 289 | **77%** | | 0.1 | **0.3** | **22.5** |
| A1-KNH | 15 | 357 | 285 | **80%** | | 0.0 | **0.4** | **18.6** |
| A1-KNH | 18 | 355 | 285 | **80%** | | 0.0 | **0.7** | **21.4** |
| A1-KNH | 24 | 344 | 270 | **78%** | | 0.0 | **0.4** | **14.8** |
| A2-TOT | 0 | 617 | 439 | **71%** | | 0.5 | **12.5** | **33.3** |
| A2-TOT | 3 | 618 | 284 | **46%** | | 0.5 | **0.4** | **1.1** |
| A2-TOT | 6 | 608 | 170 | **28%** | | 0.2 | **1.2** | **18.8** |
| A2-TOT | 9 | 611 | 271 | **44%** | | 0.4 | **3.0** | **19.6** |
| A2-TOT | 12 | 647 | 246 | **38%** | | 0.0 | **1.2** | **21.5** |
| A2-TOT | 15 | 639 | 255 | **40%** | | 1.0 | **8.6** | **30.2** |
| A2-TOT | 18 | 626 | 178 | **28%** | | 1.6 | **5.1** | **23.0** |
| A2-TOT | 24 | 679 | 460 | **68%** | | 0.4 | **2.4** | **28.0** |
| B1-TPN | 0 | 316 | 308 | **97%** | | 0.2 | **4.2** | **19.5** |
| B1-TPN | 3 | 330 | 284 | **86%** | | 0.2 | **2.1** | **16.5** |
| B1-TPN | 6 | 345 | 283 | **82%** | | 0.1 | **0.7** | **18.7** |
| B1-TPN | 9 | 366 | 318 | **87%** | | 0.1 | **0.6** | **11.0** |
| B1-TPN | 12 | 298 | 256 | **86%** | | * | **0.0** | **1.2** |
| B1-TPN | 15 | 297 | 226 | **76%** | | 1.0 | **0.9** | **2.2** |
| B1-TPN | 18 | 301 | 259 | **86%** | | 0.3 | **0.8** | **2.7** |
| B1-TPN | 24 | 312 | 239 | **77%** | | 0.0 | **0.0** | **4.2** |
| B2-HKT | 0 | 710 | 540 | **76%** | | 0.3 | **8.3** | **22.4** |
| B2-HKT | 3 | 866 | 528 | **61%** | | 0.3 | **4.4** | **21.8** |
| B2-HKT | 6 | 865 | 468 | **54%** | | 0.0 | **1.5** | **19.0** |
| B2-HKT | 9 | 928 | 641 | **69%** | | 0.0 | **1.4** | **16.1** |
| B2-HKT | 12 | 878 | 501 | **57%** | | 0.0 | **0.0** | **1.6** |
| B2-HKT | 15 | 861 | 500 | **58%** | | 0.0 | **0.2** | **10.6** |
| B2-HKT | 18 | 879 | 470 | **53%** | | 0.0 | **0.4** | **12.8** |
| B2-HKT | 24 | 916 | 561 | **61%** | | 0.0 | **0.2** | **18.2** |

| **Village** | **Month** | **N neg** | **N PV** | **% PV** | **N PF** | **% PF** | **N PF+PV** | **% PF+PV** | **N P.spp** | **% P.spp** | **N neg with fever** | **% neg with fever** | **N PV with fever** | **% PV with fever** | **N PF with fever** | **% PF with fever** | **N PF+PV with fever** | **% PF+PV with fever** | **N P.spp with fever** | **% P.spp with fever** |
| --- | --- | --- | --- | --- | --- | --- | --- | --- | --- | --- | --- | --- | --- | --- | --- | --- | --- | --- | --- | --- |
| A1-KNH | 0 | 230 | 44 | 12.2 | 57 | 15.7 | 16 | 4.4 | 15 | 4.1 | 16 | 7.0 | 2 | 4.5 | 5 | 8.8 | 1 | 6.3 | 0 | 0.0 |
| A1-KNH | 3 | 265 | 2 | 0.7 | 0 | 0.0 | 0 | 0.0 | 1 | 0.4 | 20 | 7.5 | 0 | 0.0 | 0 |  | 0 |  | 0 | 0.0 |
| A1-KNH | 6 | 217 | 33 | 13.0 | 2 | 0.8 | 0 | 0.0 | 2 | 0.8 | 24 | 11.1 | 1 | 3.0 | 0 | 0.0 | 0 |  | 1 | 50.0 |
| A1-KNH | 9 | 225 | 60 | 20.5 | 2 | 0.7 | 0 | 0.0 | 6 | 2.0 | 17 | 7.6 | 6 | 10.0 | 0 | 0.0 | 0 |  | 1 | 16.7 |
| A1-KNH | 12 | 223 | 55 | 19.0 | 0 | 0.0 | 0 | 0.0 | 11 | 3.8 | 15 | 6.7 | 3 | 5.5 | 0 |  | 0 |  | 0 | 0.0 |
| A1-KNH | 15 | 231 | 51 | 17.9 | 1 | 0.4 | 0 | 0.0 | 2 | 0.7 | 53 | 22.9 | 12 | 23.5 | 0 | 0.0 | 0 |  | 0 | 0.0 |
| A1-KNH | 18 | 222 | 52 | 18.2 | 2 | 0.7 | 0 | 0.0 | 9 | 3.2 | 13 | 5.9 | 0 | 0.0 | 0 | 0.0 | 0 |  | 0 | 0.0 |
| A1-KNH | 24 | 230 | 24 | 8.9 | 0 | 0.0 | 1 | 0.4 | 15 | 5.6 | 17 | 7.4 | 1 | 4.2 | 0 |  | 0 | 0.0 | 2 | 13.3 |
| A2-TOT | 0 | 241 | 86 | 19.6 | 23 | 5.2 | 3 | 0.7 | 86 | 19.6 | 30 | 12.4 | 16 | 18.6 | 3 | 13.0 | 2 | 66.7 | 18 | 20.9 |
| A2-TOT | 3 | 280 | 3 | 1.1 | 1 | 0.4 | 0 | 0.0 | 0 | 0.0 | 18 | 6.4 | 0 | 0.0 | 0 | 0.0 | 0 |  | 0 |  |
| A2-TOT | 6 | 137 | 30 | 17.6 | 1 | 0.6 | 1 | 0.6 | 1 | 0.6 | 11 | 8.0 | 5 | 16.7 | 0 | 0.0 | 0 | 0.0 | 0 | 0.0 |
| A2-TOT | 9 | 210 | 49 | 18.1 | 6 | 2.2 | 0 | 0.0 | 6 | 2.2 | 43 | 20.5 | 11 | 22.4 | 0 | 0.0 | 0 |  | 1 | 16.7 |
| A2-TOT | 12 | 191 | 42 | 17.1 | 2 | 0.8 | 1 | 0.4 | 10 | 4.1 | 2 | 1.0 | 1 | 2.4 | 0 | 0.0 | 0 | 0.0 | 0 | 0.0 |
| A2-TOT | 15 | 159 | 67 | 26.3 | 13 | 5.1 | 3 | 1.2 | 13 | 5.1 | 21 | 13.2 | 10 | 14.9 | 8 | 61.5 | 0 | 0.0 | 1 | 7.7 |
| A2-TOT | 18 | 128 | 38 | 21.3 | 5 | 2.8 | 0 | 0.0 | 7 | 3.9 | 15 | 11.7 | 4 | 10.5 | 4 | 80.0 | 0 |  | 1 | 14.3 |
| A2-TOT | 24 | 324 | 119 | 25.9 | 5 | 1.1 | 4 | 0.9 | 8 | 1.7 | 61 | 18.8 | 17 | 14.3 | 0 | 0.0 | 2 | 50.0 | 3 | 37.5 |
| B1-TPN | 0 | 236 | 49 | 15.9 | 10 | 3.2 | 1 | 0.3 | 12 | 3.9 | 46 | 19.5 | 7 | 14.3 | 1 | 10.0 | 0 | 0.0 | 1 | 8.3 |
| B1-TPN | 3 | 234 | 36 | 12.7 | 2 | 0.7 | 3 | 1.1 | 9 | 3.2 | 17 | 7.3 | 0 | 0.0 | 0 | 0.0 | 1 | 33.3 | 0 | 0.0 |
| B1-TPN | 6 | 229 | 49 | 17.3 | 1 | 0.4 | 0 | 0.0 | 4 | 1.4 | 28 | 12.2 | 3 | 6.1 | 0 | 0.0 | 0 |  | 0 | 0.0 |
| B1-TPN | 9 | 281 | 32 | 10.1 | 2 | 0.6 | 0 | 0.0 | 3 | 0.9 | 40 | 14.2 | 5 | 15.6 | 0 | 0.0 | 0 |  | 1 | 33.3 |
| B1-TPN | 12 | 253 | 2 | 0.8 | 0 | 0.0 | 0 | 0.0 | 1 | 0.4 | 9 | 3.6 | 0 | 0.0 | 0 |  | 0 |  | 0 | 0.0 |
| B1-TPN | 15 | 218 | 4 | 1.8 | 1 | 0.4 | 0 | 0.0 | 2 | 0.9 | 9 | 4.1 | 0 | 0.0 | 0 | 0.0 | 0 |  | 0 | 0.0 |
| B1-TPN | 18 | 251 | 5 | 1.9 | 1 | 0.4 | 0 | 0.0 | 2 | 0.8 | 8 | 3.2 | 1 | 20.0 | 0 | 0.0 | 0 |  | 0 | 0.0 |
| B1-TPN | 24 | 229 | 8 | 3.3 | 0 | 0.0 | 0 | 0.0 | 2 | 0.8 | 11 | 4.8 | 0 | 0.0 | 0 |  | 0 |  | 0 | 0.0 |
| B2-HKT | 0 | 384 | 89 | 16.5 | 28 | 5.2 | 10 | 1.9 | 29 | 5.4 | 17 | 4.4 | 6 | 6.7 | 3 | 10.7 | 1 | 10.0 | 4 | 13.8 |
| B2-HKT | 3 | 394 | 101 | 19.1 | 15 | 2.8 | 4 | 0.8 | 14 | 2.7 | 61 | 15.5 | 19 | 18.8 | 4 | 26.7 | 2 | 50.0 | 7 | 50.0 |
| B2-HKT | 6 | 373 | 71 | 15.2 | 6 | 1.3 | 1 | 0.2 | 17 | 3.6 | 70 | 18.8 | 17 | 23.9 | 0 | 0.0 | 0 | 0.0 | 1 | 5.9 |
| B2-HKT | 9 | 530 | 85 | 13.3 | 8 | 1.2 | 1 | 0.2 | 17 | 2.7 | 79 | 14.9 | 11 | 12.9 | 2 | 25.0 | 0 | 0.0 | 1 | 5.9 |
| B2-HKT | 12 | 493 | 7 | 1.4 | 0 | 0.0 | 0 | 0.0 | 1 | 0.2 | 65 | 13.2 | 2 | 28.6 | 0 |  | 0 |  | 0 | 0.0 |
| B2-HKT | 15 | 447 | 44 | 8.8 | 0 | 0.0 | 1 | 0.2 | 8 | 1.6 | 51 | 11.4 | 12 | 27.3 | 0 |  | 0 | 0.0 | 1 | 12.5 |
| B2-HKT | 18 | 408 | 49 | 10.4 | 2 | 0.4 | 0 | 0.0 | 11 | 2.3 | 94 | 23.0 | 10 | 20.4 | 0 | 0.0 | 0 |  | 0 | 0.0 |
| B2-HKT | 24 | 458 | 81 | 14.4 | 1 | 0.2 | 0 | 0.0 | 21 | 3.7 | 54 | 11.8 | 21 | 25.9 | 0 | 0.0 | 0 |  | 4 | 19.0 |

***5.3 Individual results for multiple P. falciparum infections identified during prevalence surveys by uPCR.***

**Table S4: Number of *P. falciparum* or mixed infections diagnosed by uPCR per subject over the 24-month follow-up.**

| **Number of  *P. falciparum* or mixed infections** | **Number of *P. falciparum* infected individuals** | **Individuals with consecutive infections (≤3 months apart)** | | | |
| --- | --- | --- | --- | --- | --- |
|  |  | None | 2 | 3 | 4 |
| 1 | 196 | 196 |  |  |  |
| 2 | 14 | 4 | 10 |  |  |
| 3 | 5 | 0 | 3 | 2 |  |
| 4 | 2 | 0 | 1 | 0 | 1 |
| **Total** | 217 |  |  |  |  |

There were no particular characteristics of individuals found infected several times with PF compared to general population of PF positive:

- 62% (13/21) males among recurrent positive individuals, versus 69% males among all PF positive,
- mean age 29 years among recurrent positive individuals, versus 28 years among all PF positive,
- 19% (4/21) G6PD deficient versus 23% among among all PF positive.

**Table S5: Detailed results for 21 individuals with multiple *P. falciparum* (PF) or mixed (PF+PV) positive uPCR results.** First survey after MDA is highlighted in green. *month of delayed survey: Participants who were absent since the start of MDA and came back before the end of the MDA intervention were sampled upon their first treatment uptake.

| **Village** | **Participant ID** | **N rounds of MDA** | **0** | **3** | **6** | **9** | **12** | **15** | **18** | **24** |
| --- | --- | --- | --- | --- | --- | --- | --- | --- | --- | --- |
| Intervention | 20011 | 3 | PF | Neg |  | Neg |  |  | PF |  |
| Intervention | 20054 | 3 | Neg | Neg |  | Neg | Neg |  | PF | PF |
| Intervention | 20098 | 2 | PV | Neg | PF+PV | PF |  | PF+PV |  | PF+PV |
| Intervention | 20253 | 2 | Neg (M1*) | Neg | Neg | PF | PF |  |  | P. spp |
| Intervention | 20415 | 0 |  | Neg | Neg | PF | PF |  | Neg |  |
| Intervention | 30009 | 1 | PF+PV (M1*) |  |  | PF | PV |  |  | Neg |
| Control | 10310 | 2 | PF | PF | Neg | Neg | Neg | Neg | Neg | Neg |
| Control | 40306 | 1 | PF | PF | Neg | Neg (M11*) | Neg |  |  |  |
| Control | 40310 | 0 | Neg | PF | PF |  |  |  |  |  |
| Control | 40437 | 3 | PF | PF | Neg | PF | Neg |  | Neg | Neg |
| Control | 40523 | 2 |  |  | PF | PF (M10*) | Neg |  | Neg | Neg |
| Control | 40536 | 2 | PF | PF | PF | Neg | Neg | Neg | Neg | Neg |
| Control | 40569 | 0 | PF+PV | PF |  |  |  |  |  |  |
| Control | 40645 | 0 | PF+PV | PF | Neg | Neg | Neg | Neg |  | PV |
| Control | 40655 | 0 |  |  |  |  |  |  | PF | PF |
| Control | 40669 | 3 | PV | PF+PV | PF+PV | PF | Neg | P. spp | PV | PV |
| Control | 40693 | 3 | PF | PF | Neg | PF | Neg | Neg | PV |  |
| Control | 40699 | 2 | PF+PV | PF | PF | PF | Neg | Neg | Neg | P. spp |
| Control | 40743 | 3 | PF+PV | PF |  | PV | Neg | Neg | Neg | Neg |
| Control | 40870 | 1 | PF |  | PF | PF |  |  |  |  |
| Control | 40913 | 3 |  | PF | PF | Neg | Neg |  |  |  |

***5.4 Statistical modelling using GEE: univariable analyses***

**Table S6: Univariable analysis comparing after MDA and control period to baseline.** (Corresponding to multivariable analysis in Table 3 models 1 to 4.)

|  |  | Unadjusted OR | 95%CI | p–value |
| --- | --- | --- | --- | --- |
| Age | ≤10 years | 1 | Reference | <0.0001 |
|  | >10 years | 2.8 | 2.0-4.2 |  |
| Sex | Female | 1 | Reference | <0.0001 |
|  | Male | 2.1 | 1.5-2.9 |  |
| Village | HKT | 1 | Reference | <0.0001 |
|  | TPN | 0.5 | 0.3-0.9 |  |
|  | TOT | 1.7 | 1.1-2.5 |  |
|  | KNH | 2.0 | 1.4-2.9 |  |
| Season | Cold | 1 | Reference | <0.0001 |
|  | Hot | 4.2 | 2.8-6.5 |  |
|  | Wet | 2.3 | 1.5-3.6 |  |
| Study period | Baseline | 1 | Reference | <0.0001 |
|  | Control | 0.2 | 0.1-0.3 |  |
|  | After MDA | 0.08 | 0.05–0.11 |  |
| Time*Study period | Baseline | 1 | Reference | 0.0001 |
|  | Control period | 0.5 | 0.3-0.9 |  |
|  | After MDA | 0.04 | 0.02-0.10 |  |
|  | Months in control period | 0.86 | 0.78-0.94 |  |
|  | Months after MDA | 1.04 | 1.0-1.08 |  |
| Individual  participation to  MDA | Baseline | 1 | Reference | <0.0001 |
|  | Control period | 0.2 | 0.1-0.3 |  |
|  | No MDA | 0.3 | 0.1-0.5 |  |
|  | 1 or 2 rounds | 0.10 | 0.06-0.18 |  |
|  | 3 rounds | 0.04 | 0.02-0.07 |  |
| Individual MDA  participation with  time variations | Baseline | 1 | Reference | 0.0005 |
|  | Control period | 0.5 | 0.3-0.9 |  |
|  | No MDA taken | 0.10 | 0.01-0.9 |  |
|  | 1 or 2 rounds MDA | 0.12 | 0.04-0.3 |  |
|  | 3 rounds MDA | 0.01 | 0.004-0.06 |  |
|  | Time during control period | 0.85 | 0.77-0.94 |  |
|  | Time after MDA: no MDA taken | 1.06 | 0.95-1.18 |  |
|  | Time after MDA: 1 or 2 rounds MDA | 0.99 | 0.94-1.05 |  |
|  | Time after MDA: 3 rounds MDA | 1.07 | 1.0-1.14 |  |

**Table S7: Multivariable analysis comparing after MDA and control period to baseline.**  Supplementary exploration of individual participation to MDA impact on overall risk of *P. falciparum* infection post-MDA (model 3) and on its trend after MDA (model 4)

| Variable | Categories | | Adjusted OR | 95%CI | p–value |
| --- | --- | --- | --- | --- | --- |
| Model 3. Study period (including individual MDA participation after MDA) | | Baseline (M0 survey) | 1 | Reference | <0.0001 |
|  |  | Control period | 0.4 | 0.3-0.7 |  |
|  |  | After MDA, No MDA taken | 0.26 | 0.13-0.53 |  |
|  |  | After MDA, 1 or 2 rounds MDA | 0.09 | 0.05-0.15 |  |
|  |  | After MDA, 3 rounds MDA | 0.04 | 0.02-0.08 |  |
| Model 4. Interaction between study period (including individual MDA participation after MDA)and time | Study period | Baseline (M0 survey) | 1 | Reference | <0.0001 |
|  |  | Control period | 0.4 | 0.2-0.9 |  |
|  |  | After MDA, No MDA taken | 0.06 | 0.01-0.4 |  |
|  |  | After MDA, 1 or 2 rounds MDA | 0.05 | 0.02-0.1 |  |
|  |  | After MDA, 3 rounds MDA | 0.01 | 0.003-0.03 |  |
|  | Time (for each additional month) | Control period | 1.02 | 0.9-1.2 |  |
|  |  | After MDA, No MDA taken | 1.10 | 1.03-1.17 |  |
|  |  | After MDA, 1 or 2 rounds MDA | 1.03 | 0.98-1.09 |  |
|  |  | After MDA, 3 rounds MDA | 1.08 | 0.99-1.18 |  |

**Table S8: Univariable analysis comparing after MDA and control period only.** This model includes M3 to M24 and corresponds to multivariable analysis presented in table S9.

|  |  | Unadjusted OR | [95%CI] | p–value |
| --- | --- | --- | --- | --- |
| Age | ≤10 years | 1 | Reference | 0.007 |
|  | >10 years | 2.3 | 1.3–4.3 |  |
| Sex | Female | 1 | Reference | 0.003 |
|  | Male | 2.2 | 1.3–3.8 |  |
| Village | HKT | 1 | Reference | <0.0001 |
|  | TPN | 0.5 | 0.2–1.1 |  |
|  | TOT | 2.0 | 1.1–3.5 |  |
|  | KNH | 0.3 | 0.1–0.8 |  |
| Season | Cold | 1 | Reference | 0.15 |
|  | Hot | 1.3 | 0.8–2.1 |  |
|  | Wet | 1.5 | 1.0–2.3 |  |
| Study period | Control | 1 | Reference | 0.0001 |
|  | After MDA | 0.4 | 0.3–0.6 |  |
| Baseline PF infection | No baseline PF | 1 | Reference | 0.0001 |
|  | Baseline PF | 4.5 | 2.2–9.1 |  |
|  | No baseline data | 2.3 | 1.3–4.1 |  |
| Study period# Baseline PF | Control period # no baseline PF | 1 | Reference | <0.0001 |
|  | Control period # baseline PF | 20.1 | 8.3–48.9 |  |
|  | Control period # no baseline data | 2.0 | 0.9–4.3 |  |
|  | after MDA # no baseline PF | 0.6 | 0.3–1.2 |  |
|  | after MDA # baseline PF | 0.03 | 0.01–0.2 |  |
|  | after MDA # no baseline data | 1.5 | 0.7–3.2 |  |
| Time*Study period | Control period | 1 | Reference | <0.0001 |
|  | Months in control period | 0.86 | 0.78–0.94 |  |
|  | After MDA | 0.2 | 0.1–0.3 |  |
|  | Months after MDA | 1.04 | 1.0–1.07 |  |
| Study period (including individual  participation to  MDA) | No MDA | 1 | Reference | <0.0001 |
|  | 1 or 2 rounds | 0.4 | 0.1–0.8 |  |
|  | 3 rounds | 0.2 | 0.1–0.4 |  |
|  | Before MDA | 0.7 | 0.3–1.5 |  |
| Interaction between study period (including individual  participation to  MDA) | Control period # no baseline PF | 1 | Reference | <0.0001 |
|  | Control period # baseline PF | 20.9 | 8.5-51.3 |  |
|  | Control period # no baseline data | 1.9 | 0.9-4.3 |  |
|  | After MDA: No MDA* | 2.8 | 1.2-6.6 |  |
|  | After MDA: 1 or 2 rounds* | 1.0 | 0.5-2.2 |  |
|  | After MDA: 3 rounds* | 0.4 | 0.2-0.9 |  |

* irrespective of baseline PF infection status (only 5 individuals with baseline PF infection did not take MDA)

**Table S9: Multivariable analysis comparing after MDA and control period only.** This model includes M3 to M24 and corresponds to univariable analysis presented in table S8.

| Variable | Categories | Adjusted OR | 95%CI | p–value |
| --- | --- | --- | --- | --- |
| Age | ≤10 years | 1 | Reference | 0.0492 |
|  | >10 years | 1.9 | 1.0-3.5 |  |
| Sex | Female | 1 | Reference | 0.0085 |
|  | Male | 2.1 | 1.2-3.5 |  |
| Village | B2–HKT | 1 | Reference |  |
|  | B1–TPN | 0.6 | 0.3-1.5 |  |
|  | A2–TOT | 13.0 | 4.6-36.7 |  |
|  | A1–KNH | 2.5 | 0.7-9.0 |  |
| Season | Cold | 1 | Reference | 0.0076 |
|  | Hot | 1.0 | 0.6-1.8 |  |
|  | Wet | 1.9 | 1.1-3.3 |  |
| Interaction between study period (including individual  participation to  MDA) | Control period # No baseline PF (n=763) | 1 | Reference | <0.0001 |
|  | Control period # Baseline PF (n=59) | 17.2 | 6.5–45.6 |  |
|  | Control period # No baseline data (n=498) | 2.0 | 0.8–4.9 |  |
|  | After MDA: No MDA # No baseline PF or no data* (n=222) | 0.5 | 0.1–2.3 |  |
|  | After MDA: 1 or 2 rounds # Any baseline PF status (n=861) | 0.2 | 0.1–0.6 |  |
|  | After MDA: 3 rounds # Any baseline PF status (n=871) | 0.1 | 0.03–0.4 |  |

* only 5 individuals detected with an infection during M0 did not receive any MDA, so that the OR can’t be calculated. The OR associated with after MDA period for individuals infected at baseline and no participation in MDA was estimated by combination of the other coefficients to OR [95%CI]=6.9 [1.1–42.3].

***5.5 Evolution of adjusted OR of P. falciparum infection over time (GEE multivariable analysis, table 3 model 2).***


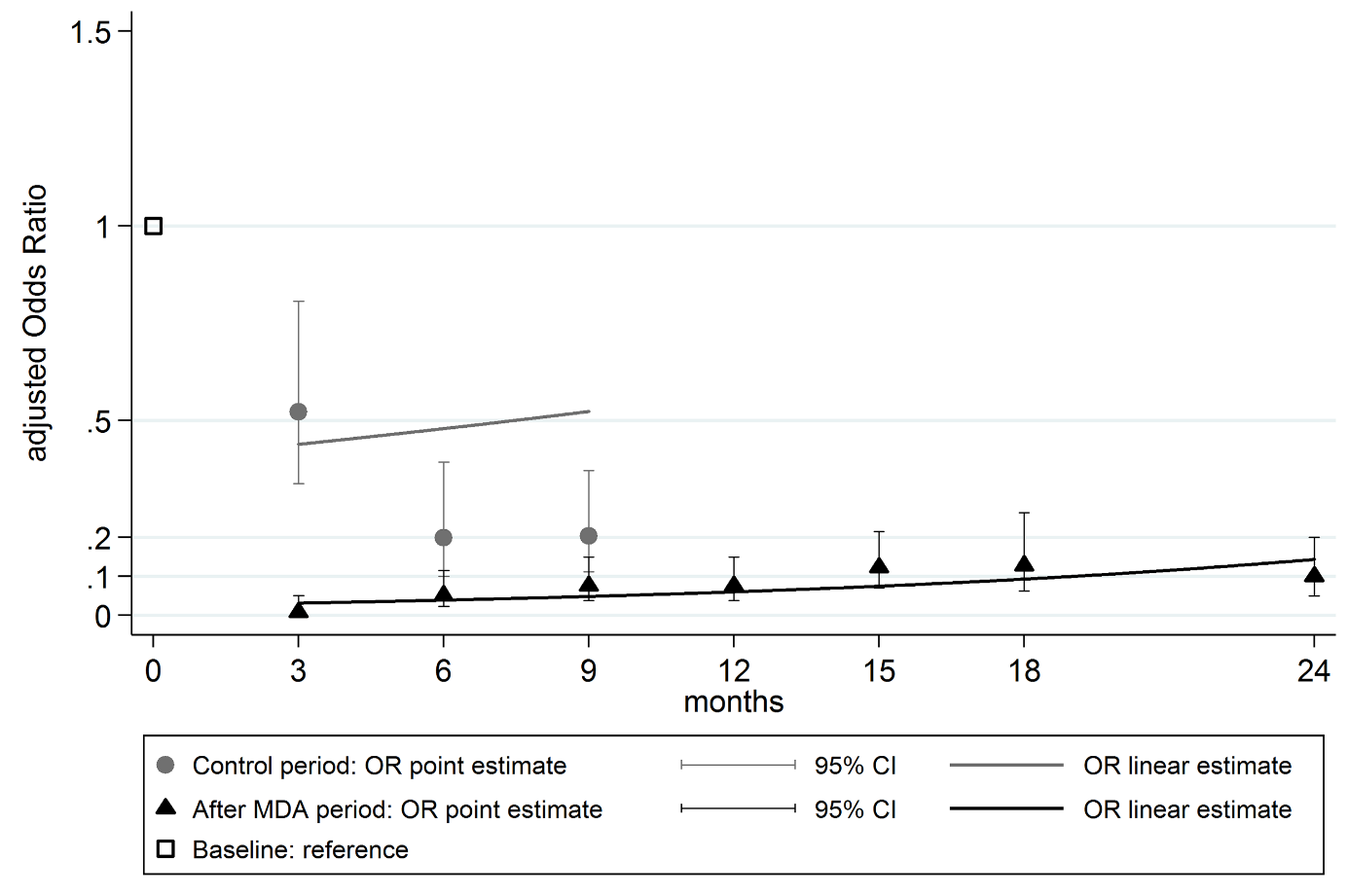


**Figure S5: Adjusted OR of *P. falciparum* carriage compared to M0 baseline, according to time.** Point estimates were obtained using month as a categorical variable and linear estimates were obtained using month as a continuous variable (as presented in table 3 model 2). Estimations were separated for before and after MDA periods.

**6. Access to early diagnosis and treatment at Malaria Post**

Weeks without any patient seen at MP were considered weeks with interrupted MP activity. Access of MP was evaluated as the mean number of consultations per 100 inhabitants per active week.

In three out of the four villages, the number of weeks without patients was <6 weeks out of over 100 weeks of follow-up. In village A2–TOT, there were 17 weeks without patients, 16 of which occurred during the first year. The average number of consultations per active week differed significantly across the 4 villages, ranging from 2.0 consultations per 100 inhabitants in A2–TOT to 3.8 consultations per 100 inhabitants in B2–HKT (p=0.0001).


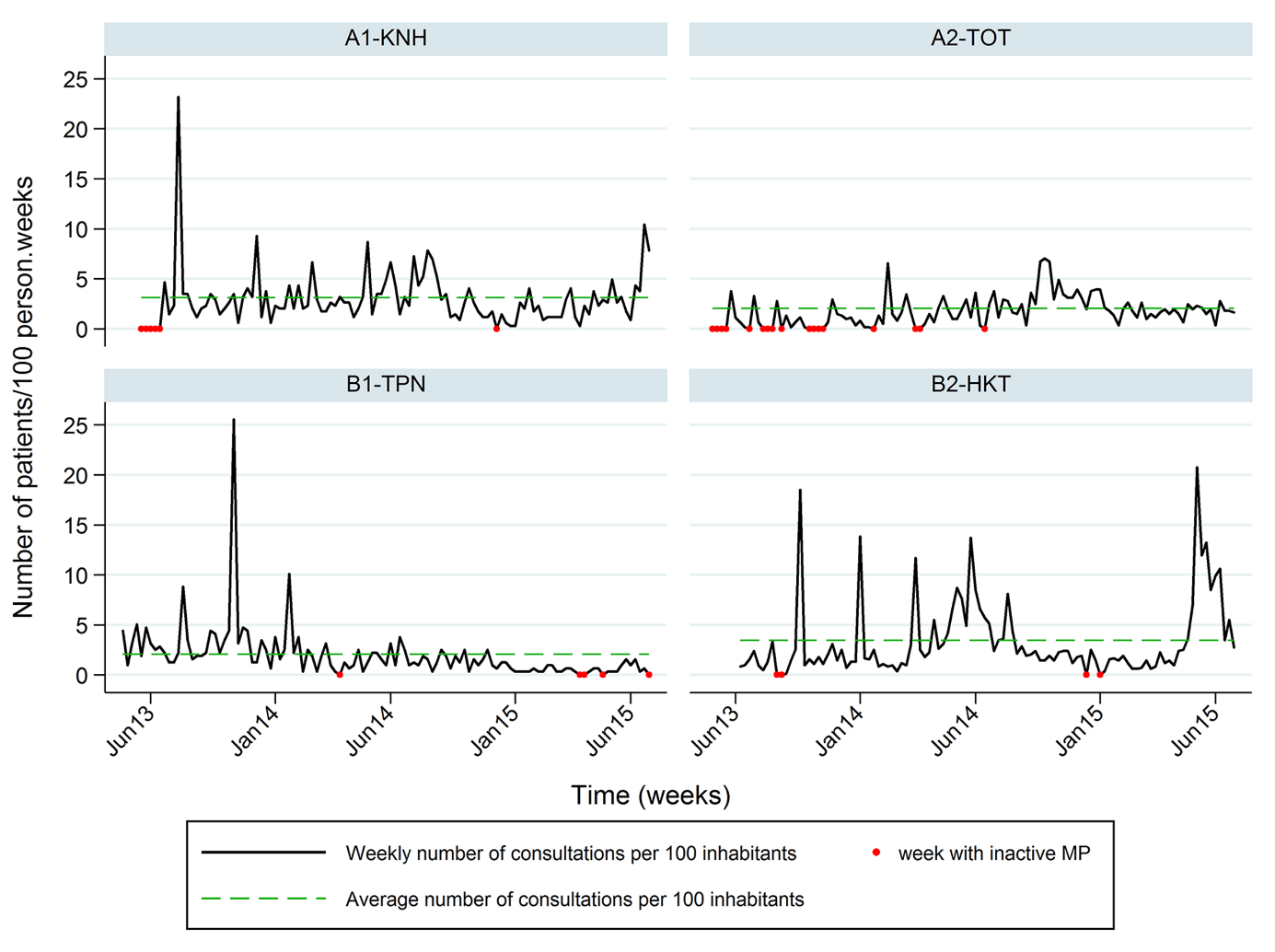


**Figure S6: Malaria Post activity in the 4 villages.** Weekly number of rapid diagnostic tests (RDT) performed, weeks without patients, and average RDT per week when active.
