# Supplementary material for: Safety and effectiveness of mass drug administration to accelerate elimination of artemisinin-resistant falciparum malaria: A pilot trial in four villages of Eastern Myanmar
Source: Wellcome Open Res. 2017 Sep 6;2:81. [Version 1] doi: 10.12688/wellcomeopenres.12240.1 (PMC5635445; doi:10.12688/wellcomeopenres.12240.1)
Supplement: Peer review history

# Peer review: Referee response for version 1

**DOI:** 10.21956/wellcomeopenres.13252.r25742

**Published:** 2017-10-09

**Authors:** Chris J. Drakeley, Bronner Goncalves, Gillian Stresman

**Affiliations:** 1 Faculty of Infectious and Tropical Diseases, London School of Hygiene & Tropical Medicine, London, UK; 2 Department of Immunology and Infection, London School of Hygiene & Tropical Medicine, London, UK; 3 Faculty of Infectious & Tropical Diseases, London School of Hygiene & Tropical Medicine, London, UK

This is a comprehensive presentation of findings from a pilot trial in which Landier and colleagues show that mass drug administration had an immediate effect on falciparum parasite carriage, in both human and vector populations. In 3 of the 4 study villages, prevalence of falciparum infection remained low during the 24 month follow-up period. The effects of MDA are particularly impressive given the fluid nature of the population and relatively low level of effective coverage (especially in larger villages). MDA appeared not to affect the incidence of clinical malaria episodes when compared to the control (‘pre-MDA’) villages. However, in the control villages, early diagnosis and treatment provided by MP was associated with reductions in malaria transmission, comparable to that seen in villages with MP and MDA. The relative contribution of MDA over MP is a key question for malaria control programmes as they seek to decide whether ensuring basic diagnostics and treatment is sufficient to eliminate malaria. The data are interesting but fall short of answering this comprehensively given the small sample size - presumably this will be a focus of the main study.

Abstract:

 It is not clear what refers to falciparum and what refers to *vivax* infections. It seems from the methods section that the criterion was >10% falciparum prevalence. Also, it is not clear by what method this prevalence is assessed.

 What population size is village size small or large?

 Vector positivity presumably means sporozoite.

Introduction:

 The evidence for the effect of treatment of the reduction of transmission is quite sparse and typically from areas with good health systems and low transmission. Context could be improved here.

 It might help to cite studies that show mosquito feeding experiments in SE Asia have shown that most infections come from symptomatic infections.

 Primaquine does not strictly sterilize infections – it has little demonstrable effect on asexual parasites.

Methods:

 Were the numbers malaria posts proportional to population?

 What was the rationale for no radical cure for *P.vivax*?

 Why was parasite prevalence was not assessed in infants? One might expect that the overall transmission effect of MDA (and MP) on parasite prevalence would also be seen in this group.

 Does malaria infection prevalence represent for each or for either ?

 Why the change in time for MDA from M12 to M9? this seems irrational and inconsistent with the rigour of the general context of the study. It does not allow similar follow up periods and confounds any potential seasonal effects.

Results:

 How did population movement relate to coverage? Were those individuals who spent less time in villages less likely to receive MDA? What was the prevalence of infection in this group?

 There is a lack of spatial analysis. The authors could use the georeferenced data to estimate whether MDA coverage predicted residual parasite prevalence spatially. Similarly, it would be interesting to understand if compliance was spatially aggregated (presumably it was). Also, were AEs related to coverage? The prevalence of AEs declined after each round, but that could be related to individuals most likely to develop AEs refusing to receive additional treatment. What was the prevalence of AEs in the different rounds in those individuals who received all 3 treatments?

 The most informative analysis is the comparison in the control villages before MDA (so effect due to MP alone) versus after MDA (combined MDA and MP effects). The GEE model estimated that the post-MDA period was associated with 5 fold reduction in falciparum carriage. However there was a clear trend of declining falciparum prevalence during the pre-MDA period. How much of this 5-fold reduction is related to the continued reduction in transmission due to MP.

 “During the pre-MDA control period, individual with baseline *P. falciparum* infections had a significantly increased odds of subsequent *P. falciparum* carriage…” – is this related to age? To occupation? Residence?

  It would be interesting to see a graph that shows temporal variation in the entomological parameters together with human prevalence and comments on the effect of seasonality.

Discussion

 The authors argue that access to diagnosis and treatment had an important effect on transmission that was “augmented by MDA”. The design of the study and the limited number of villages does not really justify this conclusion. In the control villages, where MDA was implemented 9 months after improved access to diagnosis and treatment, there was a clear trend of decreasing transmission before MDA. It is not clear how the authors expect to quantify effectiveness of MDA, that is independent of MP, using this design. This needs to be mentioned early in the discussion as this comparison is the main question the study wants to address: the added value of MDA in area with improved access to diagnosis and treatment.

 Authors could discuss what additional interventions should be considered to sustain vivax transmission reduction.

 It might be worth speculating how malaria elimination can be delayed by not treating first trimester pregnant women and infants.

We have read this submission. We believe that we have an appropriate level of expertise to confirm that it is of an acceptable scientific standard.

# Peer review: Referee response for version 1

**DOI:** 10.21956/wellcomeopenres.13252.r25746

**Published:** 2017-09-27

**Authors:** Rick M. Fairhurst

**Affiliations:** 1 Laboratory of Malaria and Vector Research, National Institute of Allergy and Infectious Diseases, National Institutes of Health , Rockville, MD, USA

Suggest line numbers be added to future manuscript versions to facilitate peer review.

 Page 3, right column, para 1, line 2: Suggest mentioning the net-to-person ratio for bed net distribution. Were all individuals provided a net, was each household provided a single net to share, etc.?

 Page 3, right column, para 2, line 8: Suggest mentioning the lower age at which assent was obtained.

 Page 5, right column, para 2: Can the authors explain why uptake was higher in the smaller versus the larger villages? Did the larger villages experience more out-migration during the study period, were there logistical problems in tracking down all previous participants in the larger villages, etc.?

 Page 5, right column, para 3, line 1: Is the acceptability of MDA simply inferred because AEs were mild or moderate? Or was a specific assessment of acceptability made?

 Page 5, right column, para 3: Suggest mentioning whether any of the AEs were treatment-limiting.

 Page 8, left column, para 1, line 1: Suggest mentioning why the other 31% of newcomers could not be included in the first survey.

 Page 8, Figure 2, panels A, B and D: The reappearance of Pv parasites within 3-6 months are obviously due to relapsing Pv from the liver, but do the authors know whether this rate of reappearance is typical following DP/MDA in this area? Also, can they speculate why there was very little Pv relapse in B1-TPN? This finding is mentioned twice in the manuscript text, but no speculation is offered.

 Page 10, right column, para 2, line 6: Is the difference in K13 mutation prevalence before and after MDA significant? Had the authors hypothesized a priori that MDA would have a selective effect on reducing the prevalence of K13-mutant infections?

 Page 10, right column, para 4, line 3: Suggest adding a citation to support the notion that these vectors are the contemporaneous primary malaria vectors in this area.

I have read this submission. I believe that I have an appropriate level of expertise to confirm that it is of an acceptable scientific standard.
